# Supplementary material for: Improving the management of musculoskeletal conditions: can an alternative approach to referral management underpinned by quality improvement and behavioural change theories offer a solution and a better patient experience? A mixed-methods study
Source: BMJ Open. 2019 Feb 9;9(2):e024710. doi: 10.1136/bmjopen-2018-024710 (PMC6398779; doi:10.1136/bmjopen-2018-024710)
Supplement: Author's manuscript [file bmjopen-2018-024710.draft_revisions.pdf]

BMJ Open

BMJ Open is committed to open peer review. As part of this commitment we make the peer review history of every article we publish publicly available.

When an article is published we post the peer reviewers' comments and the authors' responses online. We also post the versions of the paper that were used during peer review. These are the versions that the peer review comments apply to.

The versions of the paper that follow are the versions that were submitted during the peer review process. They are not the versions of record or the final published versions. They should not be cited or distributed as the published version of this manuscript.

BMJ Open is an open access journal and the full, final, typeset and author-corrected version of record of the manuscript is available on our site with no access controls, subscription charges or pay-per-view fees (http://bmjopen.bmj.com).

If you have any questions on BMJ Open's open peer review process please email editorial.bmjopen@bmj.com

BMJ Open

Improving the management of musculoskeletal conditions: can an alternative approach to referral management underpinned by quality improvement and behavioural change theories offer a solution and a better patient experience? A mixed methods study

Journal:	*BMJ Open*
Manuscript ID	bmjopen-2018-024710
Article Type:	Research
Date Submitted by the Author:	15-Jun-2018
Complete List of Authors:	Brown, Victoria; Queen Mary University of London, Barts and The London School of Medicine and Dentistry<br>Underwood, Martin; Warwick University, Warwick Medical School<br>Westwood, Olwyn; Queen Mary University of London, Barts and the London SMD<br>Morrissey, Dylan; Queen Mary University of London, Barts and The London School of Medicine and Dentistry
Keywords:	Health policy < HEALTH SERVICES ADMINISTRATION & MANAGEMENT, PRIMARY CARE, Musculoskeletal disorders < ORTHOPAEDIC & TRAUMA SURGERY, Quality in health care < HEALTH SERVICES ADMINISTRATION & MANAGEMENT, Protocols & guidelines < HEALTH SERVICES ADMINISTRATION & MANAGEMENT, Clinical audit < HEALTH SERVICES ADMINISTRATION & MANAGEMENT

SCHOLARONE™
Manuscripts

For peer review only

Title

Improving the management of musculoskeletal conditions: can an alternative approach to referral management underpinned by quality improvement and behavioural change theories offer a solution and a better patient experience? A mixed methods study

Authors

Victoria Tzortziou Brown, Martin Underwood, Olwyn Westwood, Dylan Morrissey

Blizard Institute, Barts and The London School of Medicine and Dentistry, Centre for Primary Care and Public Health, London, E1 2AT, UK Victoria Tzortziou Brown General practitioner, v.tzortzioubrown@qmul.ac.uk, Tel 0207 882 2510

Warwick Medical School, Warwick Clinical Trials Unit, Coventry, Warwickshire, CV4 7AL, UK Martin Underwood Professor

Barts and The London School of Medicine and Dentistry, Queen Mary University of London, London E1 2AD Olwyn Westwood Professor

Sports and Exercise Medicine, Barts and The London School of Medicine and Dentistry, The Royal London Hospital (Mile End), London, E1 4DG, UK Dylan Morrissey Consultant Physiotherapist and Reader

Correspondence to v.tzortzioubrown@qmul.ac.uk

Word count: 3,072

For peer review only - http://bmjopen.bmj.com/site/about/guidelines.xhtml

Abstract

Objectives: To assess whether a quality improvement-based approach to referral management can result in better musculoskeletal care within general practice.

Design: Prospective cohort study using mixed methodology including random effects meta-analysis and interrupted time series.

Setting and participants: 36 general practices in east London.

Intervention: Informed by the results of a Cochrane review on educational interventions to improve general practitioners' musculoskeletal care, we developed a multifaceted intervention, underpinned by quality improvement and behavioural change theories. It combined locally-agreed clinical pathways, feedback on referral rates, clinical audit and peer review.

Main outcome measures: Referral letter content, pathway adherence, referral rates, inter-practice variability and patient experience were evaluated before and after the intervention.

Results: Referral letter content on suspected diagnosis and prior management improved from a pooled pre-intervention proportion of 59% (95% CI = 53-65%) and 67% (95% CI = 61-73%) respectively to 77% (95% CI = 70-84%) and 81% (95% CI = 74-88%). Pathway adherence improved from a pooled pre-intervention percentage of 42% (95% CI = 35-48%) to 66% (95% CI = 57-76%). The effect was greater across all quality outcomes for practices with baseline performance below or equal to the pooled baseline performance. There were reductions in the variability and rates of orthopaedic referrals at 6, 12 and 18 months [referral rate relative effect 32% (95% CI = 14-48%), 30% (95% CI = 7-53%) and 30% (95% CI = 0-59%) respectively]. Patient rating of how well GPs explained the musculoskeletal condition improved by 29% (95% CI = 14-43%) and patient perception on the usefulness of the GP appointment improved by 24% (95% confidence interval 9-38%).

Conclusions: A quality improvement-based approach to referral management which values GPs` professionalism can result in improvements across a range of outcomes including referral quality, patient experience, referral rates and variability.

Strengths and limitations of this study

- The intervention was implemented within the constraints of clinical commissioning and rapid policy change demonstrating its feasibility and applicability within similar health systems.
- Unlike other healthcare interventions, it was developed with an underpinning behavioural theory and incorporated a number of behaviour influencing factors.
- The long follow-up on the impact on referral rates provided an opportunity to assess the sustainability of behavioural change over time.
- The evaluation incorporated a process evaluation and an outcomes evaluation which assessed the impact of the intervention across a range of outcomes including referral quality, referral rates, rate variability and patient experience.
- The intervention targeted behaviours at a number of levels and our evaluation could not identify the most cost-effective elements.

For peer review only - http://bmjopen.bmj.com/site/about/guidelines.xhtml

Introduction

A strong primary care infrastructure is central for a cost-effective health care system. (1) The pivotal role of the General Practitioner (GP) in providing person-focused care incorporating the gatekeeper function between primary, and the high-cost and intervention-heavy specialist care, can be crucial for optimal and sustainable health care delivery especially at a time of increasing demand. (2) (3) (4)

Typically 5% of GP consultations result in a specialist referral (2) (5), giving rise to approximately 13.6 million referrals in 2015-16 in England, an increase of over 18% in five years. (6) There have been reports that up to 30-40% of these may be clinically inappropriate or avoidable. (7) (8) However, the definition of 'appropriateness' is neither closely nor reliably defined (9) reflecting the multiple factors affecting referral behaviour (10) (11) (12) (13) (14) (15) and the lack of full understanding of the causes for the variation in referral rates. (16) (17) (18)

Furthermore, available evidence on the effectiveness of different referral management approaches is limited and conflicting, with similar approaches resulting in different outcomes when applied to different contexts. (19) (15) The theoretical underpinning of such initiatives is often unclear, their main focus is increasingly on reducing referral numbers and the majority of the available studies have evaluated the effect of interventions on just one aspect of a range of potential outcomes. (15) However, intermediate outcomes, such as the content of the referral, are key important markers of change that should be evaluated in order to determine where blocks to, or facilitators of, system-wide impact may be occurring (15) and to better understand the mechanisms by which interventions influence behavioural change. (20)

The aim of this study was to evaluate the impact of a theory-based, quality-improvement (QI) approach to GP musculoskeletal management and referral behaviour across a range of outcomes, including the quality of referral letters, the number and variability of referral rates and patient experience and by doing so, to address some of the evidence gaps in this important area.

Methods

Study design

We evaluated the implementation of a general practice focused intervention grounded in evidence synthesis underpinned by behavioural change theory, based on quality improvement (QI) principles and using mixed methodology. We used the MRC framework for complex interventions (21) in the design, implementation, evaluation, and piloting phases of the multi-faceted intervention. The evaluation incorporated a process evaluation guided by HM Treasury`s guidance for evaluation 'The Magenta Book', (22) and an outcomes evaluation which assessed the impact of the intervention on referral quality, referral rates, rate variability and patient experience.

Setting and participants

The intervention took place in the London Borough of Tower Hamlets which has an ethnically diverse population with high levels of deprivation and a lower healthy life expectancy than the national average. (23)

Since 2009, the 36 general practices have been organised into eight networks each serving a population of approximately 30,000-40,000 patients. All practices were included in the intervention.

Apart from the Trauma and Orthopaedics (T&O) and Rheumatology hospital outpatient service, people in Tower Hamlets also have access to a local intermediate musculoskeletal service (Clinical Assessment Service).

For peer review only - http://bmjopen.bmj.com/site/about/guidelines.xhtml

The intervention

The intervention had components targeting the main factors which influence clinical behaviour and behaviour change across the twelve domains described in the Theoretical Domains Framework (TDF). (24) This framework has been used successfully in the design of interventions aiming to change health professionals` behaviour in a number of clinical settings, including primary care. (25), (26) (27) A detailed account of all the components of the intervention, how they link to the above theoretical domain framework and the supporting theories which underpin them is presented in Table 1.

Intervention component and brief description	TDF factor(s) addressed	Supporting theories of behavioural change
**Local consensus processes:** Local clinical pathways on common musculoskeletal conditions (low back pain, shoulder pain and osteoarthritis) designed with input from multidisciplinary (MDT) teams (using the Delphi technique) summarizing guideline recommendations and availability of local services.	Knowledge, beliefs about consequences, memory and decision processes.	Theory of planned behaviour<br>Bandura`s social cognitive theory<br>Diffusion of innovation<br>Rational system theory
**Clinical audit:** Audit and reflection on current practice using standardised proformas, opportunity for team discussion of findings, identification of learning needs and agreeing ways to improve practice.	Knowledge, motivation and goals, social influences, behavioural regulation.	Theory of planned behaviour<br>Bandura`s social cognitive theory<br>Diffusion of Innovation<br>Social Influence Theory<br>Complexity theory<br>Rational system theory
**Feedback:** Monthly reports to provide ongoing feedback to clinicians on referral activity (comparative data at GP, practice, network and borough levels).	Motivation and goals, social influences, behavioural regulation.	Theory of planned behaviour<br>Bandura`s social cognitive theory<br>Diffusion of Innovation<br>Social Influence Theory<br>Rational system theory<br>Statistical Process Control theory
**Monthly educational meetings and outreach visits facilitated via local opinion leaders:** Opportunity to discuss the clinical pathways and clinical practice with peers (case-based discussions). Local opinion leaders (referral champions) in each network of practices facilitated discussions on referral decision making, best clinical practice and identification of learning needs.	Knowledge and skills, motivation and goals, social/professional role and identity, social influences, behavioural regulation.	Theory of planned behaviour<br>Bandura`s social cognitive theory<br>Diffusion of innovation<br>Social influence theory<br>Natural system theory

Table 1: Summary of intervention components and the theoretical framework underpinning them [table adapted from Table on Theoretical Support for Mental Health QuERI Antipsychotic Treatment Improvement Program (ATIP) Components and Tools (28)]

For peer review only - http://bmjopen.bmj.com/site/about/guidelines.xhtml

The intervention was part of a Network Improvement Scheme (NIS), with allocated funding at network level for the clinical audits, for hosting educational events and for clinician backfill to attend these. There were no financial incentives attached to clinical performance targets such as referral rates because such incentives may have a negative impact on the quality of care (29) and introduce conflicts of interests and ethical dilemmas. (30)

Process evaluation

The process evaluation included an assessment of how well each of the components of the intervention were implemented, including the level of GP practice participation in the clinical audit, educational meetings and outreach visits. The assessment was based on data from clinical commissioning group and provider progress reports. The degree of GP participation, and the feedback and reflections of GP practices and networks in their reports were analysed and grouped thematically. The results provided a measure of participants` interaction with the intervention.

Outcomes evaluation

Referral quality

To assess the impact on referral quality, we assessed three attributes as described by Blundell et al (31): necessity, quality of process, and destination. To determine necessity, we measured the adherence of referrals to the agreed local clinical pathways. We used the information contained in the referral letters as an indicator of quality of process. Finally, we calculated surgical conversion rates and the percentage of referrals that were seen once and discharged from the hospital without intervention and used these as indicators of the appropriateness of referral destination.

Data collection and analysis

Practices retrospectively audited consecutive musculoskeletal referrals before and after the intervention.

The data were presented descriptively and summarised using tables and charts. Additionally, random effects meta-analysis was undertaken (using Statsdirect statistical software) (32) to estimate the relative risk (RR) of the intervention effect. Each practice was taken as an individual study case and the results meta-analysed to produce a composite RR. The baseline proportion was calculated after pooling the proportions in each practice using random effects meta-analysis.

In addition, we did a subgroup analysis to assess the RR of the intervention effect depending on individual practices` baseline performance. Practices were divided into two subgroups depending on whether their performance for each outcome was equal or below the pooled baseline and the RR was calculated for each subgroup.

Referral rates

The data on referral numbers was based on National HES (Hospital Episode Statistics) extract data. Open Exeter data (a database of patient registration with NHS general practices) (33) was used to calculate the GP population list sizes.

Data analysis

We used an interrupted Time Series (ITS) analysis to evaluate the impact of the intervention on referral rates. ITS is a powerful quasi-experimental design for evaluating effects of interventions when random assignment is not feasible (34) and is well suited to initial evaluations of community interventions with greater use of this method advocated for community intervention research. (35) This design was particularly useful for capitalising on existing data on GP referrals collected at CCG level. In order to limit the risk of bias, the quality criteria for ITS design as published by Effective Practice and Organisation of Care (EPOC) Cochrane group (36) (37) were followed. Analysis using

For peer review only - http://bmjopen.bmj.com/site/about/guidelines.xhtml

segmented time series regression technique (ARIMA- autoregressive integrated moving average) was used to control for baseline level and trend (38), a common approach for evaluating policy and educational interventions. (39)

The analysis was undertaken using SPSS (Version 22, IBM). Relative effects were calculated using the predicted values from the ARIMA analysis. The seasonality of the data was tested using two-way ANOVA. If the results showed significant monthly variation, seasonal decomposition was used (IBM SPSS Statistics software, Version 22) and the seasonally adjusted series (SAS) was entered in the ARIMA analysis.

Referral rate variability

The referral rate variability amongst practices before, during, and after the intervention was calculated from the musculoskeletal referral data. To compare the variability of referral rates before and after the intervention, the Levene`s test was used.

Patient experience

Patient experience with GP management was assessed via an anonymous questionnaire. The questionnaire collected information on whether the patient was examined, whether they had physiotherapy prior to the referral, and on the level of patient satisfaction (on a 5-Likert scale) with the GP explanation and usefulness of their consultation.

Data collection and analysis

We sent the first questionnaire survey to 130 consecutive patients attending the musculoskeletal community clinic a month prior to the intervention and the follow-up questionnaire survey was disseminated to an equal number of patients a month after the intervention. The data collection and analysis were undertaken by team members blind to the study design and procedures.

Comparisons of proportions between the groups were undertaken using the Chi-square test or Fisher exact test (categorical variables).

Patient involvement

This was a professional intervention aiming to change GP behaviour. Patients were not involved in the design of the intervention. However, one of the main outcomes was patient experience because this is an important and often not adequately prioritised outcome when evaluating the effect of different referral management approaches. (19) The patient questionnaire incorporated questions from the General Practice Assessment Questionnaire (GPAQ) and was piloted with a group of patients prior to its use.

Results

Process evaluation

Clinical pathways for common MSK conditions were developed, actively disseminated to all practices and were available online. Monthly feedback on referral data comparing referral numbers at GP practice, network and borough levels was sent by the CCG to all GP practices and networks. The eight networks recruited local referral champions and all GP practices signed up to participate in the Network Improvement Scheme (NIS). All practices sent representatives to at least 8 out of the 12 network-based educational meetings which aimed to discuss referral activity and best practice on referrals. The network-based meetings were facilitated by the referral champions and focused not just on referral activity but also on referral quality and the results of the clinical audits. Out of the 36

For peer review only - http://bmjopen.bmj.com/site/about/guidelines.xhtml

practices, one did not submit their clinical audit within the specified timeframe while two submitted one of the two parts resulting in 33 (92%) complete returns.

Although a systematic evaluation of the mechanisms of change was outside the scope of this project, the end-of-year reports included practices` and networks` reflection on the process which gave some insight into how change might have occurred. Networks reported that the audits offered an opportunity for reflection on referral practice and identification of learning needs although two networks found the audits burdensome in terms of time. Practices and networks commented on the improved clinical knowledge and better awareness of local pathways and services which according to the Theory of Planned Behaviour (40) and Diffusion of Innovation (41) can facilitate behavioural change. They also commented that feedback on referral rates, the clinical audits, the role of referral champions and participation in educational meetings created opportunities for shared learning and reflection which may have led to change via the social influence theory. (42) Finally, the teamwork and collaboration as demonstrated in the feedback comments may have contributed towards a culture of learning, an important component of successful quality improvement initiatives. (43) (44)

Referral quality

Referral quality was assessed based on the information collected as part of the GP clinical audits. In total 521 musculoskeletal referral letters were audited and returned before the intervention and 463 after.

The clinical pathways covered the management of low back pain, shoulder pain and osteoarthritis of hip and knee joints. Half, 50% (259/521) of the referrals before the intervention and 49% (225/463) afterwards were for conditions covered in the above pathways. The results of these referrals were used to assess the effect of the intervention on pathway adherence.

Overall, there were statistically significant improvements across all five quality indicators (Table 2). However, the effect of the intervention was markedly larger for practices with baseline performance equal or below the pooled baseline performance (Table 2).

	**Effect of intervention**	**Pooled baseline proportion**	**Estimated post-intervention proportion**	**Effect of intervention for practices with baseline proportion equal or below the pooled baseline proportion**	**Effect of intervention for practices with baseline proportion above the pooled baseline proportion**
	RR (95% CI)	Proportion (95% CI)	Proportion (95% CI)	RR (95% CI) (n=number of practices)	RR (95% CI) (n=number of practices)
**Adherence to pathways**	1.57 (1.35-1.81)	42% (35%-48%)	66% (57%-76%)	2.09 (1.60-2.73) (n=17)	1.38 (1.16-1.64) (n=16)
**Letter information on suspected diagnosis**	1.31 (1.19-1.43)	59% (53-65%)	77% (70-84%)	1.74 (1.47-2.05) (n=14)	1.19 (1.09-1.29) (n=19)
**Letter information**	1.21 (1.11-1.31)	67% (61-73%)	81% (74-88%)	1.55 (1.35-1.78)	1.09 (1.02-1.15)

For peer review only - http://bmjopen.bmj.com/site/about/guidelines.xhtml

**on previous management**				(n=14)	(n=19)
**Conversion of T&O referrals to surgery**	1.45 (1.05-2)	43% (34-52%)	62% (58-65%)	2.39 (1.56-3.67) (n=17)	1.13 (0.87-1.46) (n=11)
**Seen once and discharged**	0.51 (1.33-1.78)	13% (10-17%)	6% (3%-9%)	0.50 (0.30-0.80) (n=19)	0.50 (0.16-1.52) (n=14)

Table 2: Effect of intervention using relative risk (RR) on the five indicators of GP referral quality

The above results are summarised in Figure 1.

Referral rates

Figure 2 presents the number of GP referrals to T&O and Rheumatology specialities per 1,000 patients per month between April 2009 and April 2016.

ARIMA analysis to establish whether there was an effect of the intervention revealed no statistically significant effect on Rheumatology referral rates but there was a significant decrease in the T&O referral rates at six, twelve and eighteen months post-intervention. The T&O results are summarised in Table 3.

	Estimate of effect	P	95% CI of estimate of effect	Relative effect
**Effect at 6 months**	-0.31	0.001	-0.48, -0.14	-32%
**Effect at 12 months**	-0.30	0.011	-0.53, -0.07	-30%
**Effect at 18 months**	-0.30	0.048	-0.59, 0.00	-30%
**Effect at 24 months**	-0.29	0.113	-0.65, 0.07	-30%
**Effect at 30 months**	-0.28	0.191	-0.71, 0.14	-28%
**Effect at 36 months**	-0.28	0.269	-0.77, 0.22	-27%

Table 3 Effect of intervention on Trauma and Orthopaedics (T&O) referral rates (using seasonality adjusted series ARIMA analysis)

Variability of referral rates amongst practices

The practice-level referral rates per 1,000 patients per year followed a normal distribution (Shapiro-Wilk test >0.05, SPSS software). Reductions in the variability of referral rates amongst practices were

For peer review only

For peer review only - http://bmjopen.bmj.com/site/about/guidelines.xhtml

shown in the two years following the intervention but these only reached statistical significance in the year after the intervention (2011-12) for T&O referrals (Levene`s test P=0.05) (Figure 3).

Patient experience

The response rate to the questionnaire was 81/130 (62%) prior to the intervention and 86/130 (66%) afterwards. All questionnaires returned were entered into the final analysis.

In terms of demographic characteristics, there were more participants from ethnic minority groups post-intervention (exact chi squared for two independent proportions, P= <0.001).

More participants rated the GP explanation of their condition as good/very good post-intervention [28/76 (37%) before versus 57/86 (66%) after, 95% CI 14-43%]. Additionally, more participants rated the usefulness of their appointment positively (good/very good) post-intervention [34/78 (43%) before versus 58/86 (67%) after, 95% CI 9-38%].

There were no changes with regards to examination rates and physiotherapy referrals before and after the intervention [74% (58/78) of the participants responded that they had been examined by the GP prior to the referral pre-intervention versus 77% (66/86) post-intervention, (95% CI -10% to 16%) and 39/78 (50%) of participants responded that they had been referred for physiotherapy prior to their referral before versus 49/86 (57%) after the intervention (95% CI -8% to 22%).

Discussion

The intervention resulted in improvements in the adherence of referrals to the clinical pathways, in the content of referral letters and in the conversion of T&O referrals to surgery. Referral rates were reduced at six, twelve and eighteen months following the introduction of the intervention. These results are consistent with the findings that Evans (2009) (45) (46) reported in her evaluation of a referral improvement project using peer-review in Torfaen and are particularly important in view of the nationally increasing trend of GP referrals to Orthopaedics which was estimated by Briggs to be 7-8% per annum, in his 2015 report entitled 'Getting it right first time'. (47)

The improvements in the quality of referrals show that an intervention with a developmental focus, aiming to facilitate reflection on clinical practice and assist clinicians to identify their individual learning needs and find their own solutions can be effective. The Network Improvement Scheme was a set of tools that acted as the enabler for clinicians to engage and was appropriate because maintenance of professional autonomy is important for GPs (48) and respect for professionalism has been shown to motivate them to engage in quality improvement activities. (49)

It could be that the dialogue generated in the outreach meetings with the referral champions addressed some of the intrinsic psychological factors that are important determinants of GP referral behaviour. Such factors include reduced tolerance of uncertainty or a perception that serious disease is more frequent. (18)

Patient factors, for example high patient expectations for a referral and anxiety about the condition also influence referral decisions. (19) The improvements recorded in patient experience are especially important in view of the fact that there was a statistically significant proportion of people from ethnic minority groups answering the post-intervention questionnaire as there is some evidence that they tend to evaluate their care more negatively, even after analyses have been adjusted for potential confounders. (50) (51) The questionnaire response rate compares very well with other questionnaire surveys in Tower Hamlets. (52)

For peer review only - http://bmjopen.bmj.com/site/about/guidelines.xhtml

The effect of the intervention was greater for practices with baseline performance below or at the pooled baseline. This is in accordance with a Cochrane systematic review assessing the effects of audit and feedback on the practice of health-care providers and patient outcomes. (53) Our findings suggest that it may be possible to increase the effect of interventions on professional practice by optimally designing and better targeting these to achieve the maximum benefit at a reduced cost.

The lack of sustainability of the effect on referral rates could be explained by Bandura`s Social Cognitive Theory (54) which highlights the importance of stimuli and reinforcement for learning and behavioural change.

The high engagement levels of GPs during the intervention are probably due to its developmental focus and its emphasis on improving the appropriateness and quality of referrals while respecting and empowering professional autonomy. (48) (55) Our findings and some evidence that similar approaches may result in improvements in other geographical areas (15) and in different medical specialities (56) suggest that the intervention may be generalizable in other settings. As the health care system changes and becomes more integrated, the development of more patient-centred care pathways and the possibilities that derive from new technological developments present opportunities for building on professionalism and further improving referral processes through inter-professional co-operation. (57) (58) (59)

Strengths and limitations

The intervention was implemented within the constraints of clinical commissioning and rapid policy change demonstrating its feasibility and applicability within similar health systems. Unlike other healthcare interventions, it was developed with an underpinning behavioural theory and analysis of its components indicated that it incorporated a number of behaviour influencing factors, including knowledge and skills, motivation and goals, social/professional role and identity, social influences and behavioural regulation. (25) Addressing the different barriers is important for the successful implementation of change. (60) The long follow-up on the impact on referral rates provided an opportunity to assess the sustainability of behavioural change over time.

One of the study`s limitations was the fact that the design was by necessity non-randomised. The effect of the intervention may be partly attributable to increased attention, focus and priority on referral behaviour. (61)

The intervention targeted behaviours at a number of levels and our evaluation could not identify the most cost-effective elements. There is conflicting evidence as to whether multifaceted interventions are more likely to be successful than single-component ones. (62) (63) Most attempts to change professional behaviour involve bundles of interventions and because of their complex nature it may not be possible to clearly assess the effectiveness of particular components. (64) A 2015 overview of systematic reviews suggested that 'interventions which contribute to normative restructuring of practice, modifying peer group norms and expectations (e.g. educational outreach) and relational restructuring, reinforcing modified peer group norms (e.g. reminders, audit and feedback), offer the best chances of success' and that 'combining such interventions is most likely to change behaviour'. (64) The concept of marginal gains has had a positive impact in sport and may offer a promising approach for enhancing healthcare professional behaviour and performance and for creating a culture of continuous improvement. (65)

For peer review only - http://bmjopen.bmj.com/site/about/guidelines.xhtml

Figure legends

Figure 1 Summary of audit results on the quality of GP referrals

Figure 2 Number of GP referrals to T&O and Rheumatology per 1,000 patients between April 2009 and April 2016 (the red arrow represents the time point of the intervention, where T&O=Trauma and Orthopaedics)

Figure 3 Multiple box and whisker plot showing the variability of practices` referral rates to Trauma and Orthopaedics (T&O)

For peer review only

For peer review only - http://bmjopen.bmj.com/site/about/guidelines.xhtml

Competing interest statement

All authors have completed the ICMJE uniform disclosure form at http://www.icmje.org/coi_disclosure.pdf and declare: no support other than the funder (Arthritis Research UK) for the submitted work; no financial relationships with any organisations that might have an interest in the submitted work in the previous three years, no other relationships or activities that could appear to have influenced the submitted work.

Contributorship statement

All authors designed the evaluation of the intervention. VTB led on the implementation, designed data collection tools, monitored data collection, wrote the statistical analysis plan, cleaned and analysed the data, and drafted and revised the paper. She is guarantor. All remaining authors contributed to the development of core ideas, the analysis plan, interpretation of the results, and the drafting of the paper. NHS Tower Hamlets Clinical Commissioning Group (CCG) implemented and funded the intervention as part of the local network improvement scheme. The clinical pathways were developed in collaboration with the Clinical Academic Group (CEG), Centre of Primary Care and Public Health, Barts and The London School of Medicine and Dentistry.

Acknowledgements

The authors would like to thank Arthritis Research UK for funding this evaluation as part of an educational grant. We would also like to thank NHS Tower Hamlets Clinical Commissioning Group (CCG), the Clinical Academic Group (CEG- Centre of Primary Care and Public Health, Barts and The London School of Medicine and Dentistry) and the GPs in Tower Hamlets for their support and participation in the study.

Funding

This work was supported by Arthritis Research UK (grant number 18678).

Ethics Committee Approval

The National Research Ethics Service (NRES) was contacted and confirmed in writing that ethical approval for the project was not required from the NHS research ethics committee (REC). Additionally, the Joint Research & Development office at Barts and The London was contacted and confirmed the same. Ethical approval was therefore granted by QMUL and permission to use the relevant data was also granted by the Governance Team of NHS North East London and The City.

Data sharing

The investigators will share data used in developing the results presented in this manuscript on request to the corresponding author.

For peer review only

For peer review only - http://bmjopen.bmj.com/site/about/guidelines.xhtml

References

1. Starfield B, Shi L. Policy Relevant Determinants of Health: An International Perspective. 2002; 60(3: 201-218).

2. Coulter A. Managing demand at the interface between primary and secondary care. 1998; 16(1974).

3. Starfield B. Is primary care essential? 1994; 344:1129-1133.

4. Franks P Clancy C, Nutting P. Gatekeeping revisited-protecting patients from overtreatment. 1992; 327: 424-29.

5. Parsons S, Ingram M, Clarke-Cornwell A, et al. The University of Manchester. Manchester: Arthritis Research UK Epidemiology Unit. [Online]; 2011 cited 2017 September 18. Available from: https://www.escholar.manchester.ac.uk/api/datastream?publicationPid=uk-ac-man-scw:123774&datastreamId=FULL-TEXT.PDF

6. NHS England. NHS inpatient elective admission events and outpatient referrals and attendances, Quarters 1-4 2015-2016. [Online]; 2016 cited 2017 February 8. Available from: https://www.england.nhs.uk/statistics/statistical-work-areas/hospital-activity/quarterly-hospital-activity/qar-data/

7. Speed C.A, Crisp AJ. Referrals to hospital-based rheumatology and orthopaedic services: seeking direction. 2005; 44(469-471).

8. Jones E, Stott NC. Avoidable referrals? Analysis of 170 consecutive referrals to secondary care. 1994; 309(576).

9. Roland MO, Porter RW, Matthews JG, et al. Improving care: a study of orthopaedic outpatient referrals. 1991; 302(6785:1124-1128).

10. Royal College of General Practitioners. [Online]; 2013 cited 2017 September 28. Available from: http://www.rcgp.org.uk/~/media/Files/Policy/A-Z-policy/The-2022-GP-Compendium-of-Evidence.ashx

11. Summerton N. Positive and negative factors in defensive medicine: a questionnaire study of general practitioners. 1995; 310.

12. Franks P, Williams GC, Zwanziger J, et al. Why do physicians vary so widely in their referral rates? 2000; 15(163).

13. Sullivan CO, Omar RZ, Ambler G, Majeed A. Case-mix and variation in specialist referrals in general practice. 2005; 55(516):529-533.

14. Kassam H, Tzortziou Brown V, O`Halloran P, et al. General practitioners' attitude to sport and exercise medicine services: a questionnaire-based survey. 2014; 90(680-684).

15. Blank L, Baxter S, Woods HB, et al. What is the evidence on interventions to manage referral from primary to specialist non-emergency care? A systematic review and logic model

For peer review only - http://bmjopen.bmj.com/site/about/guidelines.xhtml

synthesis. 2015; 3(24).

16. Wilkin D SA. Variation in general practitioners' referral rates to consultants. 1987; 37(301):350-3.

17. Sullivan CO, Omar RZ, Ambler G, et al. Case-mix and variation in specialist referrals in general practice. 2005; 55(516):529-33.

18. O'Donnell CA. Variation in GP referral rates: what can we learn from the literature? 2000; 17(6):462-71.

19. Foot C, Naylor C, Imison C. The quality of GP diagnosis and referral. [Online]; 2010 cited 2017 September 18. Available from: https://www.kingsfund.org.uk/sites/default/files/field/field_document/quality-gp-diagnosis-referral-gq-inquiry-research-paper-mar11.pdf

20. Pawson R, Greenhalgh J, Brennan C. Demand management for planned care: a realist synthesis. 2016; 4(2).

21. Craig P, Dieppe P, MacIntyre S, et al. Developing and evaluating complex interventions: new guidance 2008. [Online] cited 2017 October 2. Available from: www.mrc.ac.uk/complexinterventionsguidance

22. HM Treasury. The Magenta Book: guidance for evaluation. [Online]; 2011 cited 2017 October 5. Available from: https://www.gov.uk/government/uploads/system/uploads/attachment_data/file/220542/magenta_book_combined.pdf

23. TowerHamlets.gov.uk. [Online]; 2016 cited 2018 February 26. Available from: https://www.towerhamlets.gov.uk/Documents/Public-Health/JSNA/JSNA_Summary.pdf

24. Michie S JM, Abraham C, Lawton R, et al. "Psychological Theory" Group: Making psychological theory useful for implementing evidence-based practice: a consensus approach. 2005; 14(1):26-33.

25. French SD, Green SE, O'Connor DA, et al. Developing theory-informed behaviour change interventions to implement evidence into practice: a systematic approach using the Theoretical Domains Framework. 2012; 7(38).

26. Porcheret M, Main C, Croft P, et al. Development of a behaviour change intervention: a case study on the practical application of theory. 2014; 9(1:42).

27. Thomas S, Mackintosh S. Use of the theoretical domains framework to develop an intervention to improve physical therapist management of the risk of falls after discharge. 2014; 94(11:1660-75).

28. Sales A, Smith J, Curran G, Kochevar L. Models, strategies, and tools. Theory in implementing evidence-based findings into health care practice. 2006; 21(Suppl 2: S43-9).

29. Roland M. Can primary care reform reduce demand on hospital outpatient departments? Research summary 2007. [Online]; 2007 cited 2017 September 18.

For peer review only - http://bmjopen.bmj.com/site/about/guidelines.xhtml

http://www.netscc.ac.uk/hsdr/files/project/SDO_RS_08-1518-082_V01.pdf

30. Imison C, Naylor C. The Kings Fund. [Online]; 2010 cited 2017 February 8. Available from: https://www.kingsfund.org.uk/sites/files/kf/Referral-management-lessons-for-success-Candace-Imison-Chris-Naylor-Kings-Fund-August2010.pdf

31. Blundell N, Clarke A, Mays N. Interpretations of referral appropriateness by senior health managers in five PCT areas in England: a qualitative investigation. 2010; 19(3: 182-186).

32. Statsdirect statistical software. [Online]. cited 2017 October 9. http://www.statsdirect.co.uk/

33. NHS Digital. [Online]. cited 2018 February 26. Available from: https://digital.nhs.uk/NHAIS/open-exeter

34. Taljaard M, McKenzie JE, Ramsay CR, Grimshaw JM. The use of segmented regression in analysing interrupted time series studies: an example in pre-hospital ambulance care. 2014; 9(1).

35. Craig R. Interrupted Time Series Designs in health technology assessment: lessons from two systematic reviews of behaviour change strategies. 2003; 19(4:613–23).

36. Cochrane Effective Practice and Organisation of Care Group (EPOC). [Online] cited 2017 October 9. Available from: http://epoc.cochrane.org/sites/epoc.cochrane.org/files/uploads/datacollectionchecklist.pdf

37. Cochrane Effective Practice and Organisation of Care (EPOC). [Online]; 2017 cited 2017 October 9. Available from: http://epoc.cochrane.org/epoc-specific-resources-review-authors

38. Serumaga B. Effect of pay for performance on the management and outcomes of hypertension in the United Kingdom: interrupted time series study. 2011; 342.

39. Wagner AK, Soumerai S, Zhang F et al. Segmented regression analysis of interrupted time series studies in medication use research. 2002; 27(4: 299-309).

40. Ajzen I. The theory of planned behavior. 1991; 50(2:179-211) (doi:10.1016/0749-5978(91)90020-T).

41. Rogers E. Diffusion of Innovations. 5th Revised edition (17 Nov. 2003).

42. Mittman BS, Tonesk X, Jacobson PD. Implementing clinical practice guidelines: social influence strategies and practitioner behaviour change. 1992; 18(12: 413-422).

43. Plsek PE, Greenhalgh T. The challenge of complexity in health care. 2001; 323:625.

44. Dawda P, Jenkins R, Varnam R. Quality improvement in General Practice: An Enquiry into the Quality of General Practice in England. [Online]; 2010 cited 2017 October 20. Available from: https://www.kingsfund.org.uk/sites/default/files/field/field_document/quality-

For peer review only - http://bmjopen.bmj.com/site/about/guidelines.xhtml

improvement-gp-inquiry-discussion-paper-mar11.pdf

45. Evans E. The Torfaen referral evaluation project. 2009; 17(423-429).

46. Evans E, Aiking H, Edwards A. Reducing variation in general practitioner referral rates through clinical engagement and peer review of referrals: a service improvement project. 2011; 19(263-272).

47. Briggs T. A national review of adult elective orthopaedic services in England: Getting it right first time. [Online]; 2015 cited 2017 November 10. Available from: https://www.boa.ac.uk/wp-content/uploads/2015/03/GIRFT-National-Report-MarN.pdf

48. Marjoribanks T, Lewis J. Reform and autonomy: perceptions of the Australian general practice community. 2003; 56(10: 2229-2239).

49. Spooner A, Chapple A, Roland M. What makes British general practitioners take part in a quality improvement scheme? 2001; 6(3: 145-150).

50. Mead N, Roland M. Understanding why some ethnic minority patients evaluate medical care more negatively than white patients: a cross sectional analysis of a routine patient survey in English general practices. 2009; 339.

51. Campbell J, Ramsay J, Green J. Age, gender, socioeconomic and ethnic differences in patients' assessments of primary health care. 2001; 10(90-95).

52. Choudhury Y, Bremner SA, Ali A, et al. Prevalence and impact of chronic widespread pain in the Bangladeshi and White populations of Tower Hamlets, East London. 2013; 32((9):1375-1382).

53. Jamtvedt G, Young J, Kristoffersen DT, et al. Audit and feedback: effects on professional practice and health care outcomes. 2003; 3(CD000259).

54. Bandura A. Self-efficacy: toward a unifying theory of behavioural change. 1977; 84(2: 191-215).

55. Watkins C, Timm A, Gooberman-Hill R, et al. Factors affecting feasibility and acceptability of a practice- based educational intervention to support evidence-based prescribing: a qualitative study. 2004; 661.

56. Wright J, Harrison S, McGeorge M et al. Improving the management and referral of patients with transient ischaemic attacks: a change strategy for a health community. 2006; 15(1:9-12).

57. V. Tzortziou Brown, I. Patel, N. Thomas, et al. New ways of working; delivering better care for people with long-term conditions. 2017; 9(5).

58. Gilburt H. Supporting integration through new roles and working across boundaries. [Online]: The Kings Fund; 2016 cited 2017 November 13. Available from: https://www.kingsfund.org.uk/sites/default/files/field/field_publication_file/Supporting_integration_web.pdf

For peer review only - http://bmjopen.bmj.com/site/about/guidelines.xhtml

59. Martin GP, Kocman D, Stephens T, et al. Pathways to professionalism? Quality improvement, care pathways, and the interplay of standardisation and clinical autonomy. 2017.

60. Grol R, Grimshaw J. From best evidence to best practice: effective implementation of change in patients' care. 2003; 362((9391):1225–30).

61. Sedgwick P. The Hawthorne effect. 2012; 344: d8262.

62. Squires JE, Sullivan K, Eccles MP, et al. Are multifaceted interventions more effective than single-component interventions in changing health-care professionals' behaviours? An overview of systematic reviews. 2014; 9(152).

63. Ivers N, Jamtvedt G, Flottorp S, et al. Audit and feedback: effects on professional practice and healthcare outcomes. 2012;(6).

64. Johnson MJ, May CR. Promoting professional behaviour change in healthcare: what interventions work, and why? A theory-led overview of systematic reviews. 2015;(5: e008592. doi: 10.1136/bmjopen-2015-008592).

65. Hall D, James D, Marsden N. Marginal gains: Olympic lessons in high performance for organisations. HR Bulletin: Research and Practice. 2012 Nov;7(2):9-13.

For peer review only

For peer review only - http://bmjopen.bmj.com/site/about/guidelines.xhtml

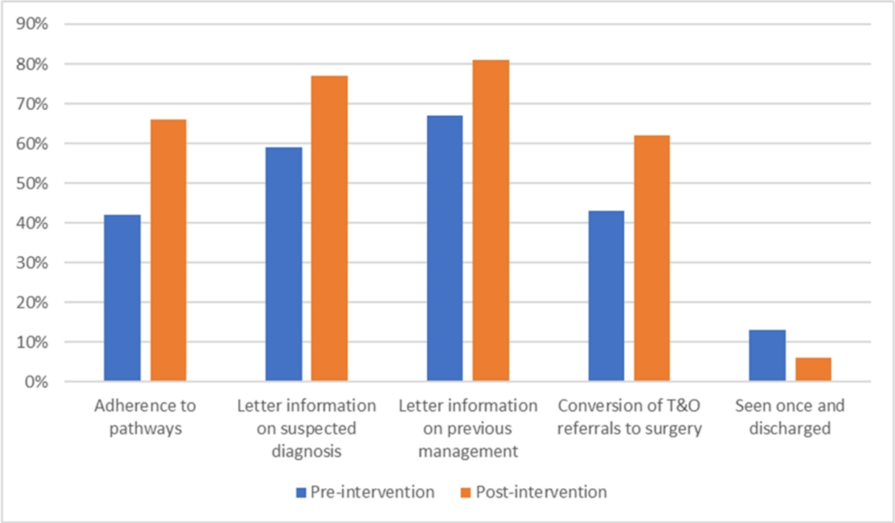

Summary of audit results on the quality of GP referrals

127x71mm (300 x 300 DPI)

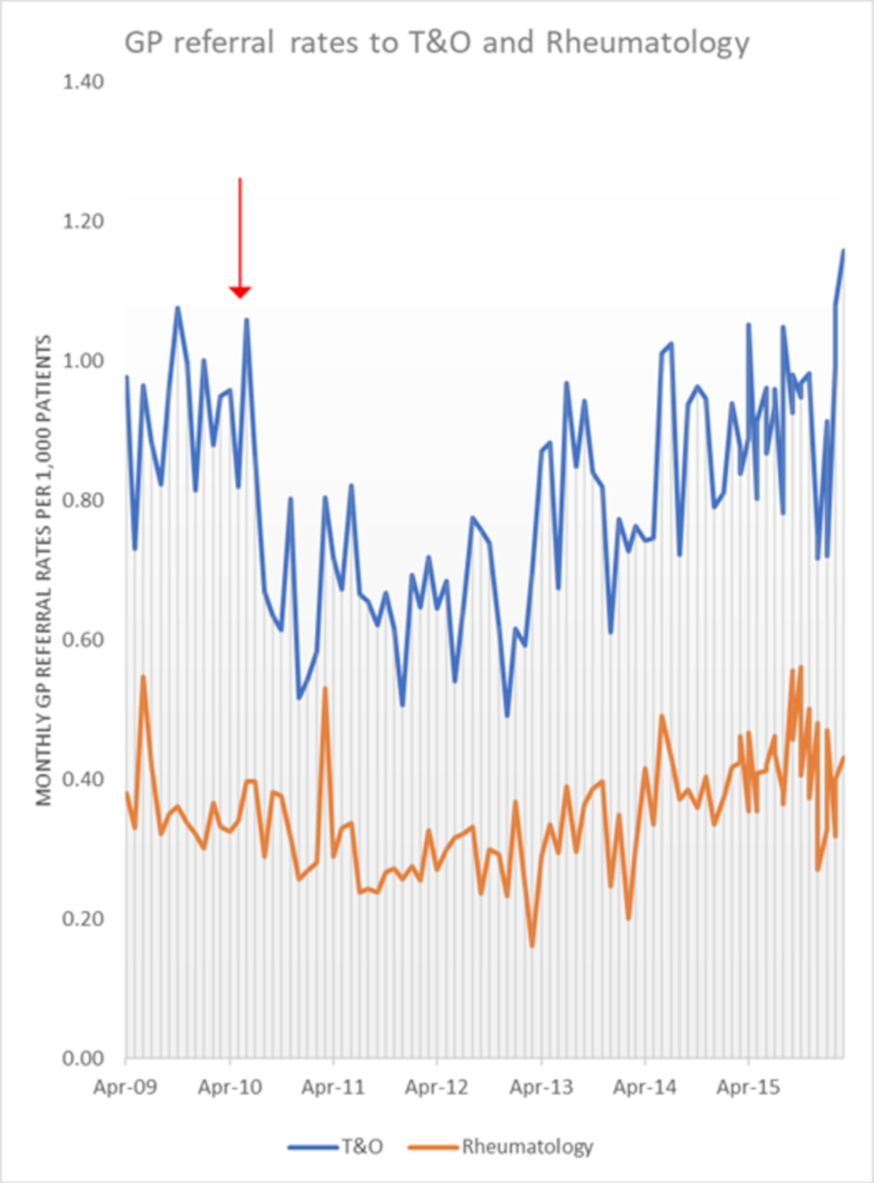

Number of GP referrals to T&O and Rheumatology per 1,000 patients between April 2009 and April 2016 (the red arrow represents the time point of the intervention, where T&O=Trauma and Orthopaedics)

139x190mm (300 x 300 DPI)

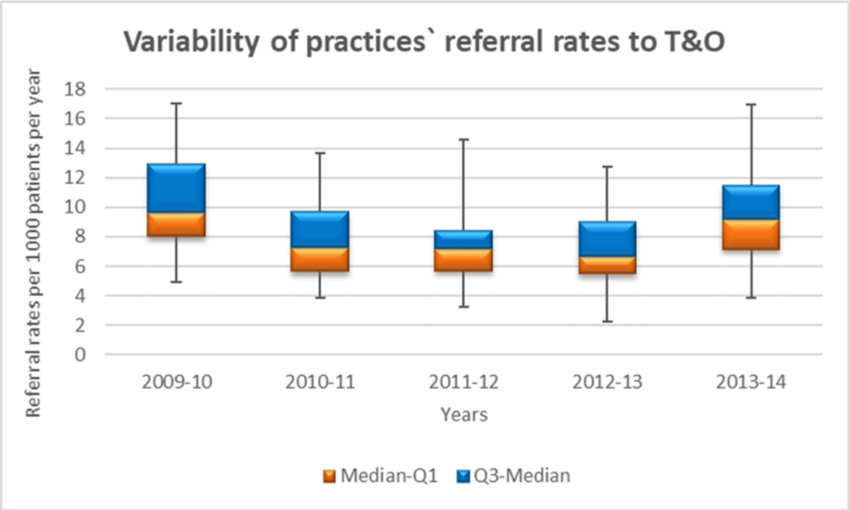

Multiple box and whisker plot showing the variability of practices` referral rates to Trauma and Orthopaedics (T&O)

127x71mm (300 x 300 DPI)

Revised Standards for Quality Improvement Reporting Excellence (SQUIRE 2.0) September 15, 2015
Text Section and Item Name Section or Item Description

	Check	Page
1. Title<br>Indicate that the manuscript concerns an initiative to improve healthcare (broadly defined to include the quality, safety, effectiveness, patient centeredness, timeliness, cost, efficiency, and equity of healthcare)	☑	1
The title of the manuscript is: "Improving the management of musculoskeletal conditions: can an alternative approach to referral management underpinned by quality improvement and behavioural change theories offer a solution and a better patient experience? A mixed methods study"		
2. Abstract<br>a. Provide adequate information to aid in searching and indexing b. Summarize all key information from various sections of the text using the abstract format of the intended publication or a structured summary such as: background, local problem, methods, interventions, results, conclusions.	☑	2
We have attempted to summarise the work in a succinct way in the abstract to assist with searching and indexing. We used the structure proposed in BMJ Open and included the objectives, design, setting and participants, intervention, main outcome measures, results and conclusion.		
3. Introduction<br>Why did you start? Problem Description Nature and significance of the local problem	☑	3
The significance of the topic has been justified in the introduction highlighting both the burden of MSK conditions and the challenges from increasing demand and number of outpatient referrals.		
4. Available knowledge Summary of what is currently known about the problem, including relevant previous studies	☑	3
The existing evidence has been summarised in the introduction and the gaps in evidence have been highlighted (e.g. limited theoretical underpinning of published interventions and insufficient evidence on effect on intermediate outcomes).		
5. Rationale<br>Informal or formal frameworks, models, concepts, and/or theories used to explain the problem, any reasons or assumptions that were used to develop the intervention(s), and reasons why the intervention(s) was expected to work	☑	4
The intervention had components targeting the main factors which influence clinical behaviour and behaviour change across the twelve domains described in the Theoretical Domains Framework (TDF). A detailed account of all the components of the intervention, how they link to the above theoretical domain framework and the supporting theories which underpin them has been presented in Table 1.		

For peer review only

6. Specific aims Purpose of the project and of this report<br>Methods What did you do?	☑	3-6
The aims and methodology of the project have been detailed in the manuscript (mixed methods including interrupted time series). We evaluated the implementation of a general practice focused intervention grounded in evidence synthesis underpinned by behavioural change theory, based on quality improvement (QI) principles and using mixed methodology. We used the MRC framework for complex interventions in the design, implementation, evaluation, and piloting phases of the multi-faceted intervention. The evaluation incorporated a process evaluation guided by HM Treasury`s guidance for evaluation 'The Magenta Book', and an outcomes evaluation which assessed the impact of the intervention on referral quality, referral rates, rate variability and patient experience.		
7. Context Contextual elements considered important at the outset of introducing the intervention(s)	☑	3-4
The context of the intervention has been described. Information has been provided on the setting of the intervention and the local availability of musculoskeletal service provision. The intervention was commissioned by the local Clinical Commissioning Group.		
8. Intervention(s)<br>a. Description of the intervention(s) in sufficient detail that others could reproduce it<br>b. Specifics of the team involved in the work	☑	4
The different elements of the intervention and the behavioural theories underpinning these have been described.<br>The intervention was co-ordinated by the local Clinical Commissioning Group. The principal investigator worked with clinical commissioners to collate and communicate all relevant referral data to practices at monthly intervals.		
9. Study of the Intervention(s)<br>a. Approach chosen for assessing the impact of the intervention(s)<br>b. Approach used to establish whether the observed outcomes were due to the intervention(s)	☑	5
A range of outcomes have been assessed and this is a strength of this study compared to other published reports which only focused on one or two relevant outcomes. The process evaluation included an assessment of how well each of the components of the intervention were implemented, including the level of GP practice participation in the clinical audit, educational meetings and outreach visits. The assessment was based on data from the clinical commissioning group and provider progress reports. The degree of GP participation, and the feedback and reflections of GP practices and networks in their reports were analysed and grouped thematically. The results provided a measure of participants` interaction with the intervention.		
10. Measures<br>a. Measures chosen for studying processes and outcomes of the intervention(s), including rationale for choosing them, their operational definitions, and their validity	☑	5-6

For peer review only

and reliability<br>b. Description of the approach to the ongoing assessment of contextual elements that contributed to the success, failure, efficiency, and cost<br>c. Methods employed for assessing completeness and accuracy of data		
The choice of measures, data collection and analysis as well as potential bias sources and limitations have been described. These are detailed in pages 5 and 6. For example with regards to the choice of measures on the quality of referrals we wrote: "To assess the impact on referral quality, we assessed three attributes as described by Blundell et al (31): necessity, quality of process, and destination. To determine necessity, we measured the adherence of referrals to the agreed local clinical pathways. We used the information contained in the referral letters as an indicator of quality of process. Finally, we calculated surgical conversion rates and the percentage of referrals that were seen once and discharged from the hospital without intervention and used these as indicators of the appropriateness of referral destination."<br>The process evaluation assisted towards an ongoing assessment of contextual elements.<br>The audit and patient questionnaires were piloted prior to implementation. All audit data from participating practices was entered in the meta-analysis and the assessment on quality was blinded against a pre-specified set of objective criteria. All outcomes on referral quality were reported.<br>The data on referral rates was collected by an independent source (National Hospital Episode Statistics extract data) which was blind to the intervention. The seasonality of the data was tested using two-way ANOVA. If the results showed significant monthly variation, seasonal decomposition was used (IBM SPSS Statistics Version 22 software). The seasonally adjusted series (SAS) was entered in the ARIMA analysis.		
11. Analysis<br>a. Qualitative and quantitative methods used to draw inferences from the data<br>b. Methods for understanding variation within the data, including the effects of time as a variable	☑	5-6
These have been described in the methodology section. For example, with regards to the analysis of referral rates we wrote:<br>"We used an interrupted Time Series (ITS) analysis to evaluate the impact of the intervention on referral rates. ITS is a powerful quasi-experimental design for evaluating effects of interventions when random assignment is not feasible and is well suited to initial evaluations of community interventions with greater use of this method advocated for community intervention research. This design was particularly useful for capitalising on existing data on GP referrals collected at CCG level. In order to limit the risk of bias, the quality criteria for ITS design as published by Effective Practice and Organisation of Care (EPOC) Cochrane group were followed. Analysis using segmented time series regression technique (ARIMA- autoregressive integrated moving average) was used to control for baseline level and trend, a common approach for evaluating policy and educational interventions.<br><br>The analysis was undertaken using SPSS (Version 22, IBM). Relative effects were calculated using the predicted values from the ARIMA analysis. The seasonality of the data was tested using two-way ANOVA. If the results showed significant monthly variation, seasonal decomposition was used (IBM SPSS Statistics software, Version		

For peer review only

22) and the seasonally adjusted series (SAS) was entered in the ARIMA analysis.”		
12. Ethical Considerations<br>Ethical aspects of implementing and studying the intervention(s) and how they were addressed, including, but not limited to, formal ethics review and potential conflict(s) of interest	☑	11
The study was considered by the relevant ethic committees and we have submitted information on the relevant approvals obtained.<br>“The National Research Ethics Service (NRES) was contacted and confirmed in writing that ethical approval for the project was not required from the NHS research ethics committee (REC). Additionally, the Joint Research & Development office at Barts and The London was contacted and confirmed the same. Ethical approval was therefore granted by QMUL and permission to use the relevant data was also granted by the Governance Team of NHS North East London and The City.”		
13. Results<br>a. Initial steps of the intervention(s) and their evolution over time (e.g., time-line diagram, flow chart, or table), including modifications made to the intervention during the project b. Details of the process measures and outcome c. Contextual elements that interacted with the intervention(s) d. Observed associations between outcomes, interventions, and relevant contextual elements e. Unintended consequences such as unexpected benefits, problems, failures, or costs associated with the intervention(s). f. Details about missing data	☑	6-9
The results have been summarised across all outcomes. No modifications were made to the intervention over time. A process evaluation took place and was reported. The contextual elements were reported as part of the process evaluation. Missing data was reported.		
14. Summary a. Key findings, including relevance to the rationale and specific aims b. Particular strengths of the project	☑	9-10
The key findings and strengths of the study have been presented under Discussion and Strengths and Limitations of the study. The main strengths and limitations have also been summarised in page 2.		
15. Interpretation<br>a. Nature of the association between the intervention(s) and the outcomes<br>b. Comparison of results with findings from other publications<br>c. Impact of the project on people and systems<br>d. Reasons for any differences between observed and anticipated outcomes, including the influence of context<br>e. Costs and strategic trade-offs, including opportunity costs	☑	9-10
The association between the intervention and the outcomes was explored using the relevant behavioural theories underpinning the intervention. Comparisons of the findings of this study with existing literature have been made in the discussion section. The impact of the project on participants was reported as part of the process		

For peer review only

evaluation. The lack of sustainability of the effect on referral rates could be explained by Bandura`s Social Cognitive Theory which highlights the importance of stimuli and reinforcement for learning and behavioural change. A cost-effectiveness or cost analysis was not undertaken.		
16. Limitations<br>a. Limits to the generalizability of the work<br>b. Factors that might have limited internal validity such as confounding, bias, or imprecision in the design, methods, measurement, or analysis<br>c. Efforts made to minimize and adjust for limitations	☑	3,10
The limitations of the study have been articulated and reflected upon.<br>For example, the last section mentions: “One of the study`s limitations was the fact that the design was by necessity non-randomised. The effect of the intervention may be partly attributable to increased attention, focus and priority on referral behaviour.” The methodology section expanded on the choice of the design and the reasons for this choice as well as the attempts made to address confounding and bias.		
17. Conclusions<br>a. Usefulness of the work<br>b. Sustainability<br>c. Potential for spread to other contexts<br>d. Implications for practice and for further study in the field<br>e. Suggested next steps<br>Other information	☑	10
The usefulness of the work, generalisability and implications of the study have been summarised in the conclusions. The sustainability of the intervention was explored as the study had a long follow up rate. The potential next steps are summarised in the last paragraph. The developing more integrated systems of care and the technological advances offer opportunities for building on professionalism and further improving referral processes through inter-professional co-operation.		
18. Funding<br>Sources of funding that supported this work. Role, if any, of the funding organization in the design, implementation, interpretation, and reporting	☑	11
The funder (Arthritis Research UK) has been mentioned.		

For peer review only

BMJ Open

Improving the management of musculoskeletal conditions: can an alternative approach to referral management underpinned by quality improvement and behavioural change theories offer a solution and a better patient experience? A mixed methods study

Journal:	*BMJ Open*
Manuscript ID	bmjopen-2018-024710.R1
Article Type:	Research
Date Submitted by the Author:	20-Nov-2018
Complete List of Authors:	Tzortziou Brown, Victoria; Queen Mary University of London, Barts and The London School of Medicine and Dentistry<br>Underwood, Martin; Warwick University, Warwick Medical School; University Hospitals of Coventry And Warwickshire<br>Westwood, Olwyn; Queen Mary University of London, Barts and the London SMD<br>Morrissey, Dylan; Queen Mary University of London, Barts and The London School of Medicine and Dentistry
**Primary Subject Heading**:	General practice / Family practice
Secondary Subject Heading:	Health policy, Evidence based practice, Health services research
Keywords:	Health policy < HEALTH SERVICES ADMINISTRATION & MANAGEMENT, PRIMARY CARE, Musculoskeletal disorders < ORTHOPAEDIC & TRAUMA SURGERY, Quality in health care < HEALTH SERVICES ADMINISTRATION & MANAGEMENT, Protocols & guidelines < HEALTH SERVICES ADMINISTRATION & MANAGEMENT, Clinical audit < HEALTH SERVICES ADMINISTRATION & MANAGEMENT

SCHOLARONE™
Manuscripts

Title

Improving the management of musculoskeletal conditions: can an alternative approach to referral management underpinned by quality improvement and behavioural change theories offer a solution and a better patient experience? A mixed methods study

Authors

Victoria Tzortziou Brown, Martin Underwood, Olwyn Westwood, Dylan Morrissey

Author affiliations

Blizard Institute, Barts and The London School of Medicine and Dentistry, Centre for Primary Care and Public Health, London, E1 2AT, UK **Victoria Tzortziou Brown General Practitioner**, v.tzortzioubrown@qmul.ac.uk, Tel 0207 882 2510

Warwick Medical School, Warwick Clinical Trials Unit, Coventry, Warwickshire, CV4 7AL, UK and University Hospitals of Coventry And Warwickshire, Clifford Bridge Road, Coventry CV2 2DX **Martin Underwood Professor**

Barts and The London School of Medicine and Dentistry, Queen Mary University of London, London E1 2AD **Olwyn Westwood Professor**

Sports and Exercise Medicine, Barts and The London School of Medicine and Dentistry, The Royal London Hospital (Mile End), London, E1 4DG, UK **Dylan Morrissey Professor of Sports and Musculoskeletal Physiotherapy**

Correspondence to v.tzortzioubrown@qmul.ac.uk

Word count: 4,998

For peer review only

Abstract

Objectives: To assess whether a quality improvement-based approach to referral management can result in better musculoskeletal care within general practice.

Design: Prospective cohort study using mixed methodology including random effects meta-analysis and interrupted time series.

Setting and participants: 36 general practices in east London.

Intervention: Informed by the results of a Cochrane review on educational interventions to improve general practitioners' musculoskeletal care, we developed a multifaceted intervention, underpinned by quality improvement and behavioural change theories. It combined locally-agreed clinical pathways, feedback on referral rates, clinical audit and peer review.

Main outcome measures: Referral letter content, pathway adherence, referral rates, inter-practice variability and patient experience were evaluated before and after the intervention.

Results: Referral letter content on suspected diagnosis and prior management improved from a pooled pre-intervention proportion of 59% (95% CI = 53-65%) and 67% (95% CI = 61-73%) respectively to 77% (95% CI = 70-84%) and 81% (95% CI = 74-88%). Pathway adherence improved from a pooled pre-intervention percentage of 42% (95% CI = 35-48%) to 66% (95% CI = 57-76%). The effect was greater across all quality outcomes for practices with baseline performance below or equal to the pooled baseline performance. There were reductions in the variability and rates of orthopaedic referrals at 6, 12 and 18 months [referral rate relative effect 32% (95% CI = 14-48%), 30% (95% CI = 7-53%) and 30% (95% CI = 0-59%) respectively]. Patient rating of how well GPs explained the musculoskeletal condition improved by 29% (95% CI = 14-43%) and patient perception on the usefulness of the GP appointment improved by 24% (95% confidence interval 9-38%).

Conclusions: A quality improvement-based approach to referral management which values GPs` professionalism can result in improvements across a range of outcomes including referral quality, patient experience, referral rates and variability.

Strengths and limitations of this study

- The intervention was implemented within the constraints of clinical commissioning and rapid policy change demonstrating its feasibility and applicability within similar health systems.
- Unlike other healthcare interventions, it was developed with an underpinning behavioural theory and incorporated a number of behaviour influencing factors.
- The long follow-up on the impact on referral rates provided an opportunity to assess the sustainability of behavioural change over time.
- The evaluation incorporated a process evaluation and an outcomes evaluation which assessed the impact of the intervention across a range of outcomes including referral quality, referral rates, rate variability and patient experience.
- The intervention targeted behaviours at a number of levels and our evaluation could not identify the most cost-effective elements.

For peer review only - http://bmjopen.bmj.com/site/about/guidelines.xhtml

For peer review only

Introduction

A strong primary care infrastructure is central for a cost-effective health care system. (1) The pivotal role of the General Practitioner (GP) in providing person-focused care incorporating the gatekeeper function between primary, and the high-cost and intervention-heavy specialist care, can be crucial for optimal and sustainable health care delivery especially at a time of increasing demand. (2) (3) (4)

Typically 5% of GP consultations result in a specialist referral (2) (5), giving rise to approximately 13.6 million referrals in 2015-16 in England, an increase of over 18% in five years. (6) There have been reports that up to 30-40% of these may be clinically inappropriate or avoidable. (7) (8) However, the definition of 'appropriateness' is neither closely nor reliably defined (9) reflecting the multiple factors affecting referral behaviour (10) (11) (12) (13) (14) (15) and the lack of full understanding of the causes for the variation in referral rates. (13) (16) (17)

Furthermore, available evidence on the effectiveness of different referral management approaches is limited and conflicting, with similar approaches resulting in different outcomes when applied to different contexts. (18) (15) The theoretical underpinning of such initiatives is often unclear, their main focus is increasingly on reducing referral numbers and the majority of the available studies have evaluated the effect of interventions on just one aspect of a range of potential outcomes. (15) However, intermediate outcomes, such as the content of the referral, are key important markers of change that should be evaluated in order to determine where blocks to, or facilitators of, system-wide impact may be occurring (15) and to better understand the mechanisms by which interventions influence behavioural change. (19)

The aim of this study was to evaluate the impact of a theory-based, quality-improvement (QI) approach to GP musculoskeletal management and referral behaviour across a range of outcomes, including the quality of referral letters, the number and variability of referral rates and patient experience and by doing so, to address some of the evidence gaps in this important area.

Methods

Study design

We evaluated the implementation of a general practice focused intervention grounded in evidence synthesis underpinned by behavioural change theory, based on quality improvement (QI) principles and using mixed methodology. We used the MRC framework for complex interventions (20) in the design, implementation, evaluation, and piloting phases of the multi-faceted intervention. The evaluation incorporated a process evaluation guided by HM Treasury`s guidance for evaluation 'The Magenta Book', (21) and an outcomes evaluation which assessed the impact of the intervention on referral quality, referral rates, rate variability and patient experience.

Setting and participants

The intervention took place in the London Borough of Tower Hamlets which has an ethnically diverse population with high levels of deprivation and a lower healthy life expectancy than the national average. (22)

Since 2009, the 36 general practices have been organised into eight networks each serving a population of approximately 30,000-40,000 patients. All practices were included in the intervention.

For peer review only - http://bmjopen.bmj.com/site/about/guidelines.xhtml

Apart from the Trauma and Orthopaedics (T&O) and Rheumatology hospital outpatient service, people in Tower Hamlets also have access to a local intermediate musculoskeletal service (Clinical Assessment Service).

The intervention

The intervention had components targeting the main factors which influence clinical behaviour and behaviour change across the twelve domains described in the Theoretical Domains Framework (TDF). (23) This framework has been used successfully in the design of interventions aiming to change health professionals` behaviour in a number of clinical settings, including primary care. (24), (25) (26) A detailed account of all the components of the intervention, how they link to the above theoretical domain framework and the supporting theories which underpin them is presented in Table 1 [table adapted from Table on Theoretical Support for Mental Health QuERI Antipsychotic Treatment Improvement Program (ATIP) Components and Tools (27)].

Intervention component and brief description	TDF factor(s) addressed	Supporting theories of behavioural change
**Local consensus processes:** Local clinical pathways on common musculoskeletal conditions (low back pain, shoulder pain and osteoarthritis) designed with input from multidisciplinary (MDT) teams (using the Delphi technique) summarizing guideline recommendations and availability of local services. These pathways were disseminated to all 36 practices, were presented at local educational events and were published on the local website for GPs to access.	Knowledge, beliefs about consequences, memory and decision processes.	Theory of planned behaviour<br>Bandura`s social cognitive theory<br>Diffusion of innovation<br>Rational system theory
**Clinical audit:** Audit and reflection on current practice using standardised proformas, opportunity for team discussion of findings, identification of learning needs and agreeing ways to improve practice. The audits took place at practice level and the results were discussed both within practices and also at network events.	Knowledge, motivation and goals, social influences, behavioural regulation.	Theory of planned behaviour<br>Bandura`s social cognitive theory<br>Diffusion of Innovation<br>Social Influence Theory<br>Complexity theory<br>Rational system theory
**Feedback:** Monthly reports to provide ongoing feedback to clinicians on referral activity (comparative data at GP, practice, network and borough levels). These reports were disseminated both at practice and network levels.	Motivation and goals, social influences, behavioural regulation.	Theory of planned behaviour<br>Bandura`s social cognitive theory<br>Diffusion of Innovation<br>Social Influence Theory<br>Rational system theory<br>Statistical Process Control theory
**Monthly educational meetings and outreach visits facilitated via local opinion leaders:** Opportunity to discuss the clinical pathways and clinical practice with peers (case-based discussions). Local opinion	Knowledge and skills, motivation and goals, social/professional role and identity, social influences, behavioural regulation.	Theory of planned behaviour<br>Bandura`s social cognitive theory<br>Diffusion of innovation<br>Social influence theory

For peer review only - http://bmjopen.bmj.com/site/about/guidelines.xhtml

leaders (referral champions) in each network of practices facilitated discussions on referral decision making, best clinical practice and identification of learning needs. The meetings were attended by GPs and practice managers from each practice.	Natural system theory

Table 1: Summary of intervention components and the theoretical framework underpinning them

The intervention was part of the Tower Hamlets Clinical Commissioning Group (CCG) Network Improvement Scheme (NIS), which acted as the system driver providing the incentives for change while ensuring a rigorous, structured approach incorporating planned objectives within set timelines over a year`s period. The CCG`s project management team ensured that the intervention would be delivered at scale and pace. The NIS offered allocated funding at network level for the clinical audits, for hosting educational events and for clinician backfill to attend these. There were no financial incentives attached to clinical performance targets such as referral rates because such incentives may have a negative impact on the quality of care (28) and introduce conflicts of interests and ethical dilemmas. (29)

Process evaluation

The process evaluation included an assessment of how well each of the components of the intervention were implemented, including the level of GP practice participation in the clinical audit, educational meetings and outreach visits. The assessment was based on data from CCG and provider progress reports. The degree of GP participation, and the feedback and reflections of GP practices and networks in their reports were analysed and grouped thematically. The results provided a measure of participants` interaction with the intervention.

Outcomes evaluation

Referral quality

To assess the impact on referral quality, we assessed three attributes as described by Blundell et al (30): necessity, quality of process, and destination. To determine necessity, we measured the adherence of referrals to the agreed local clinical pathways on back pain, shoulder or osteoarthritis (provided that the reason for the referral was one of the above conditions). The pathways covered all the aspects of management within primary care including the indications for investigations, medication, physiotherapy, the role of steroid injections and provided resources on exercise and advice for patients. The information on the referral letter and EMIS notes was compared with the relevant pathway during the audit process. The EMIS records were used in addition to the information contained in the referral letters in order to ensure that all relevant information on the prior management of patients was captured. We recognised that a simple yes/no answer could be subjective and might not reliably capture adherence to the clinical pathway. Therefore, the audit proforma incorporated questions on the specific management of the patient prior to the referral and on what could have been done differently according to the pathway. We used the information contained in the referral letters as an indicator of quality of process. Finally, we calculated surgical conversion rates and the percentage of referrals that were seen once and discharged from the hospital without intervention and used these as indicators of the appropriateness of referral destination.

For peer review only - http://bmjopen.bmj.com/site/about/guidelines.xhtml

Data collection and analysis

Practices retrospectively audited consecutive musculoskeletal referrals before and after the intervention.

The data were presented descriptively and summarised using tables and charts. Additionally, random effects meta-analysis was undertaken (using Statsdirect statistical software) (31) to estimate the relative risk (RR) of the intervention effect. Each practice was taken as an individual study case and the results meta-analysed to produce a composite RR. The baseline proportion was calculated after pooling the proportions in each practice using random effects meta-analysis.

In addition, we did a subgroup analysis to assess the RR of the intervention effect depending on individual practices` baseline performance. Practices were divided into two subgroups depending on whether their performance for each outcome was equal or below the pooled baseline and the RR was calculated for each subgroup.

Referral rates

The data on referral numbers was based on National HES (Hospital Episode Statistics) extract data. Open Exeter data (a database of patient registration with NHS general practices) (32) was used to calculate the GP population list sizes.

Data analysis

We used an interrupted Time Series (ITS) analysis to evaluate the impact of the intervention on referral rates. ITS is a powerful quasi-experimental design for evaluating effects of interventions when random assignment is not feasible (33) and is well suited to initial evaluations of community interventions with greater use of this method advocated for community intervention research. (34) This design was particularly useful for capitalising on existing data on GP referrals collected at CCG level. In order to limit the risk of bias, the quality criteria for ITS design as published by Effective Practice and Organisation of Care (EPOC) Cochrane group (35) (36) were followed. Analysis using segmented time series regression technique (ARIMA- autoregressive integrated moving average) was used to control for baseline level and trend (37), a common approach for evaluating policy and educational interventions. (38)

The analysis was undertaken using SPSS (Version 22, IBM). Relative effects were calculated using the predicted values from the ARIMA analysis. The seasonality of the data was tested using two-way ANOVA. If the results showed significant monthly variation, seasonal decomposition was used (IBM SPSS Statistics software, Version 22) and the seasonally adjusted series (SAS) was entered in the ARIMA analysis.

Referral rate variability

The referral rate variability amongst practices before, during, and after the intervention was calculated from the musculoskeletal referral data. To compare the variability of referral rates before and after the intervention, the Levene`s test was used.

Patient experience

Patient experience with GP management was assessed via an anonymous questionnaire. The questionnaire collected information on whether the patient was examined, whether they had physiotherapy prior to the referral, and on the level of patient satisfaction (on a 5-Likert scale) with the GP explanation and usefulness of their consultation.

For peer review only - http://bmjopen.bmj.com/site/about/guidelines.xhtml

Data collection and analysis

We sent the first questionnaire survey to 130 consecutive patients attending the musculoskeletal community clinic a month prior to the intervention and the follow-up questionnaire survey was disseminated to an equal number of patients a month after the intervention. The data collection and analysis were undertaken by team members blind to the study design and procedures.

Comparisons of proportions between the groups were undertaken using the Chi-square test or Fisher exact test (categorical variables).

Patient involvement

This was a professional intervention aiming to change GP behaviour. Patients were not involved in the design of the intervention. However, one of the main outcomes was patient experience because this is an important and often not adequately prioritised outcome when evaluating the effect of different referral management approaches. (19) The patient questionnaire incorporated questions from the General Practice Assessment Questionnaire (GPAQ) and was piloted with a group of patients prior to its use.

Results

Process evaluation

Clinical pathways for common MSK conditions were developed, actively disseminated to all practices and were available online. Monthly feedback on referral data comparing referral numbers at GP practice, network and borough levels was sent by the CCG to all GP practices and networks. The eight networks recruited local referral champions and all GP practices signed up to participate in the Network Improvement Scheme (NIS). All practices sent representatives to at least 8 out of the 12 network-based educational meetings which aimed to discuss referral activity and best practice on referrals. The network-based meetings were facilitated by the referral champions and focused not just on referral activity but also on referral quality and the results of the clinical audits. Out of the 36 practices, one did not submit their clinical audit within the specified timeframe while two submitted one of the two parts resulting in 33 (92%) complete returns.

Although a systematic evaluation of the mechanisms of change was outside the scope of this project, the end-of-year reports included practices` and networks` reflection on the process which gave some insight into how change might have occurred. Networks reported that the audits offered an opportunity for reflection on referral practice and identification of learning needs although two networks found the audits burdensome in terms of time. Practices and networks commented on the improved clinical knowledge and better awareness of local pathways and services which according to the Theory of Planned Behaviour (39) and Diffusion of Innovation (40) can facilitate behavioural change. They also commented that feedback on referral rates, the clinical audits, the role of referral champions and participation in educational meetings created opportunities for shared learning and reflection which may have led to change via the social influence theory. (41) Finally, the teamwork and collaboration as demonstrated in the feedback comments may have contributed towards a culture of learning, an important component of successful quality improvement initiatives. (42) (43)

For peer review only

For peer review only - http://bmjopen.bmj.com/site/about/guidelines.xhtml

Referral quality

Referral quality was assessed based on the information collected as part of the GP clinical audits. In total 521 musculoskeletal referral letters were audited and returned before the intervention and 463 after.

The clinical pathways covered the management of low back pain, shoulder pain and osteoarthritis of hip and knee joints. Half, 50% (259/521) of the referrals before the intervention and 49% (225/463) afterwards were for conditions covered in the above pathways. The results of these referrals were used to assess the effect of the intervention on pathway adherence.

Overall, there were statistically significant improvements across all five quality indicators (Table 2). However, the effect of the intervention was markedly larger for practices with baseline performance equal or below the pooled baseline performance (Table 2).

	Effect of intervention	Pooled baseline proportion	Estimated post-intervention proportion	Effect of intervention for practices with baseline proportion equal or below the pooled baseline proportion	Effect of intervention for practices with baseline proportion above the pooled baseline proportion
	RR (95% CI)	Proportion (95% CI)	Proportion (95% CI)	RR (95% CI) (n=number of practices)	RR (95% CI) (n=number of practices)
**Adherence to pathways**	1.57 (1.35-1.81)	42% (35%-48%)	66% (57%-76%)	2.09 (1.60-2.73) (n=17)	1.38 (1.16-1.64) (n=16)
**Letter information on suspected diagnosis**	1.31 (1.19-1.43)	59% (53-65%)	77% (70-84%)	1.74 (1.47-2.05) (n=14)	1.19 (1.09-1.29) (n=19)
**Letter information on previous management**	1.21 (1.11-1.31)	67% (61-73%)	81% (74-88%)	1.55 (1.35-1.78) (n=14)	1.09 (1.02-1.15) (n=19)
**Conversion of T&O referrals to surgery**	1.45 (1.05-2)	43% (34-52%)	62% (58-65%)	2.39 (1.56-3.67) (n=17)	1.13 (0.87-1.46) (n=11)
**Seen once and discharged**	0.51 (1.33-1.78)	13% (10-17%)	6% (3%-9%)	0.50 (0.30-0.80) (n=19)	0.50 (0.16-1.52) (n=14)

Table 2: Effect of intervention using relative risk (RR) on the five indicators of GP referral quality

The above results are summarised in Figure 1.

For peer review only - http://bmjopen.bmj.com/site/about/guidelines.xhtml

Referral rates

Figure 2 presents the number of GP referrals to T&O and Rheumatology specialities per 1,000 patients per month between April 2009 and April 2016.

Two-way ANOVA showed significant seasonality of the data for T&O referrals and therefore the seasonally adjusted series (SAS) was used for the analysis.

ARIMA analysis to establish whether there was an effect of the intervention revealed no statistically significant effect on Rheumatology referral rates but there was a significant decrease in the T&O referral rates at six, twelve and eighteen months post-intervention. The T&O results are summarised in Table 3.

	Estimate of effect	P	95% CI of estimate of effect	Relative effect
**Effect at 6 months**	-0.31	0.001	-0.48, -0.14	-32%
**Effect at 12 months**	-0.30	0.011	-0.53, -0.07	-30%
**Effect at 18 months**	-0.30	0.048	-0.59, 0.00	-30%
**Effect at 24 months**	-0.29	0.113	-0.65, 0.07	-30%
**Effect at 30 months**	-0.28	0.191	-0.71, 0.14	-28%
**Effect at 36 months**	-0.28	0.269	-0.77, 0.22	-27%

Table 3 Effect of intervention on Trauma and Orthopaedics (T&O) referral rates (using seasonality adjusted series ARIMA analysis)

For peer review only

Variability of referral rates amongst practices

The practice-level referral rates per 1,000 patients per year followed a normal distribution (Shapiro-Wilk test >0.05, SPSS software). Reductions in the variability of referral rates amongst practices were shown in the two years following the intervention but these only reached statistical significance in the year after the intervention (2011-12) for T&O referrals (Levene`s test P=0.05) (Figure 3).

Patient experience

The response rate to the questionnaire was 81/130 (62%) prior to the intervention and 86/130 (66%) afterwards. All questionnaires returned were entered into the final analysis.

In terms of demographic characteristics, there were more participants from ethnic minority groups post-intervention (exact chi squared for two independent proportions, P= <0.001).

For peer review only - http://bmjopen.bmj.com/site/about/guidelines.xhtml

More participants rated the GP explanation of their condition as good/very good post-intervention [28/76 (37%) before versus 57/86 (66%) after, 95% CI 14-43%]. Additionally, more participants rated the usefulness of their appointment positively (good/very good) post-intervention [34/78 (43%) before versus 58/86 (67%) after, 95% CI 9-38%].

There were no changes with regards to examination rates and physiotherapy referrals before and after the intervention [74% (58/78) of the participants responded that they had been examined by the GP prior to the referral pre-intervention versus 77% (66/86) post-intervention, (95% CI -10% to 16%) and 39/78 (50%) of participants responded that they had been referred for physiotherapy prior to their referral before versus 49/86 (57%) after the intervention (95% CI -8% to 22%).

Discussion

The intervention resulted in improvements in the adherence of referrals to the clinical pathways, in the content of referral letters and in the conversion of T&O referrals to surgery. Referral rates were reduced at six, twelve and eighteen months following the introduction of the intervention. These results are consistent with the findings that Evans (2009) (44) (45) reported in her evaluation of a referral improvement project using peer-review in Torfaen and are particularly important in view of the nationally increasing trend of GP referrals to Orthopaedics which was estimated by Briggs to be 7-8% per annum, in his 2015 report entitled 'Getting it right first time'. (46)

Despite the observed actual reduction in rheumatology referral rates, this did not reach statistical significance according to the interrupted time series analysis. This might be explained by the fact that the clinical pathways that were disseminated as part of the intervention mainly addressed conditions that were traditionally referred to T&O rather than rheumatology, such as radicular back pain and hip/knee osteoarthritis. Additionally, the simplicity of the message that patients who do not need or who would not consider surgery should not be referred to a surgeon, which was highlighted in the clinical pathways, may have assisted towards reducing T&O referrals. In contrast, referrals to rheumatology require more complex considerations, including assessing the possibility of inflammatory pathology, which are more challenging to address in a brief clinical pathway which may explain the smaller effect on the number of rheumatology referrals.

The improvements in the quality of referrals show that an intervention with a developmental focus, aiming to facilitate reflection on clinical practice and assist clinicians to identify their individual learning needs and find their own solutions can be effective. The Network Improvement Scheme was a set of tools that acted as the enabler for clinicians to engage and was appropriate because maintenance of professional autonomy is important for GPs (47) and respect for professionalism has been shown to motivate them to engage in quality improvement activities. (48)

It could be that the dialogue generated in the outreach meetings with the referral champions addressed some of the intrinsic psychological factors that are important determinants of GP referral behaviour. Such factors include reduced tolerance of uncertainty or a perception that serious disease is more frequent. (17)

Patient factors, for example high patient expectations for a referral and anxiety about the condition also influence referral decisions. (18) The improvements recorded in patient experience are especially important in view of the fact that there was a statistically significant proportion of people from ethnic minority groups answering the post-intervention questionnaire as there is some evidence that they tend to evaluate their care more negatively, even after analyses have been adjusted for potential

For peer review only - http://bmjopen.bmj.com/site/about/guidelines.xhtml

confounders. (49) (50) The questionnaire response rate compares very well with other questionnaire surveys in Tower Hamlets. (51)

The effect of the intervention was greater for practices with baseline performance below or at the pooled baseline. This is in accordance with a Cochrane systematic review assessing the effects of audit and feedback on the practice of health-care providers and patient outcomes. (52) Our findings suggest that it may be possible to increase the effect of interventions on professional practice by optimally designing and better targeting these to achieve the maximum benefit at a reduced cost.

The lack of sustainability of the effect on referral rates could be explained by Bandura`s Social Cognitive Theory (53) which highlights the importance of stimuli and reinforcement for learning and behavioural change. Because of the turnover of clinical staff and the competing priorities within general practice it may be important that components of the intervention which act as stimuli for reflection, such as the clinical audit and the facilitated peer-review meetings, are incorporated within clinical practice for sustainable behaviour change.

The high engagement levels of GPs during the intervention are probably due to its developmental focus and its emphasis on improving the appropriateness and quality of referrals while respecting and empowering professional autonomy. (47) (54) Our findings and some evidence that similar approaches may result in improvements in other geographical areas (15) and in different medical specialities (55) suggest that the intervention may be generalizable in other settings. As the health care system changes and becomes more integrated, the development of more patient-centred care pathways and the possibilities that derive from new technological developments present opportunities for building on professionalism and further improving referral processes through inter-professional co-operation. (56) (57) (58)

Strengths and limitations

The intervention was implemented within the constraints of clinical commissioning and rapid policy change demonstrating its feasibility and applicability within similar health systems. Unlike other healthcare interventions, it was developed with an underpinning behavioural theory and analysis of its components indicated that it incorporated a number of behaviour influencing factors, including knowledge and skills, motivation and goals, social/professional role and identity, social influences and behavioural regulation. (24) Addressing the different barriers is important for the successful implementation of change. (59) The long follow-up on the impact on referral rates provided an opportunity to assess the sustainability of behavioural change over time.

One of the study`s limitations was the fact that the design was by necessity non-randomised. The effect of the intervention may be partly attributable to increased attention, focus and priority on referral behaviour. (60)

A cost-effectiveness analysis would have been useful as such information can assist commissioning and health policy decisions on referral management interventions. Such decisions need to be based on cost-effectiveness data which will allow programme assessments to be made depending on the degree that interventions maximize health for the available resources and provide the highest 'value for money'. However, a cost-effectiveness analysis requires data from multiple sources in order to allow an estimation of the impact to the whole health care system rather than one part of it. It also requires data on patient-related outcomes which can be challenging to obtain as this is not routinely collected within primary care settings.

For peer review only - http://bmjopen.bmj.com/site/about/guidelines.xhtml

The intervention targeted behaviours at a number of levels and our evaluation could not identify the most cost-effective elements. There is conflicting evidence as to whether multifaceted interventions are more likely to be successful than single-component ones. (61) (62) Most attempts to change professional behaviour involve bundles of interventions and because of their complex nature it may not be possible to clearly assess the effectiveness of particular components. (63) A 2015 overview of systematic reviews suggested that 'interventions which contribute to normative restructuring of practice, modifying peer group norms and expectations (e.g. educational outreach) and relational restructuring, reinforcing modified peer group norms (e.g. reminders, audit and feedback), offer the best chances of success' and that 'combining such interventions is most likely to change behaviour'. (63) The concept of marginal gains has had a positive impact in sport and may offer a promising approach for enhancing healthcare professional behaviour and performance and for creating a culture of continuous improvement. (64)

Conclusion

Our study shows that a quality improvement-based approach to referral management which values GPs` professionalism can result in improvements across a range of outcomes including referral quality, patient experience, referral rates and variability. The intervention is feasible, well received by GPs and can be incorporated into every day clinical practice. Targeting the intervention where baseline performance is low may yield a greater effect.

For peer review only

Figure legends

Figure 1 Summary of audit results on the quality of GP referrals

Figure 2 Number of GP referrals to T&O and Rheumatology per 1,000 patients between April 2009 and April 2016 (the red arrow represents the time point of the intervention, where T&O=Trauma and Orthopaedics)

Figure 3 Multiple box and whisker plot showing the variability of practices` referral rates to Trauma and Orthopaedics (T&O)

For peer review only

For peer review only - http://bmjopen.bmj.com/site/about/guidelines.xhtml

Competing interest statement

All authors have completed the ICMJE uniform disclosure form at http://www.icmje.org/coi_disclosure.pdf. All authors apart from Professor Underwood declare: no support other than the funder (Arthritis Research UK) for the submitted work; no financial relationships with any organisations that might have an interest in the submitted work in the previous three years, no other relationships or activities that could appear to have influenced the submitted work. Professor Underwood declares the following: he was Chair of the NICE accreditation advisory committee until March 2017 for which he received a fee. He is chief investigator or co-investigator on multiple previous and current research grants from the UK National Institute for Health Research, Arthritis Research UK and is a co-investigator on grants funded by the Australian NHMRC. He has received travel expenses for speaking at conferences from the professional organisations hosting the conferences. He is a director and shareholder of Clinvivo Ltd that provides electronic data collection for health services research. He is part of an academic partnership with Serco Ltd related to return to work initiatives. He is a co-investigator on a study receiving support in kind from Orthospace Ltd. He is an editor of the NIHR journal series for which he receives a fee.

Contributorship statement

All authors (VTB, MU, OW and DM) designed the evaluation of the intervention. VTB led on the implementation, designed data collection tools, monitored data collection, wrote the statistical analysis plan, cleaned and analysed the data, and drafted and revised the paper. She is guarantor. All remaining authors (MU, OW, DM) contributed to the development of core ideas, the analysis plan, interpretation of the results, and the drafting of the paper. NHS Tower Hamlets Clinical Commissioning Group (CCG) implemented and funded the intervention as part of the local network improvement scheme. The clinical pathways were developed in collaboration with the Clinical Academic Group (CEG), Centre of Primary Care and Public Health, Barts and The London School of Medicine and Dentistry.

Acknowledgements

The authors would like to thank Arthritis Research UK for funding this evaluation as part of an educational grant. We would also like to thank NHS Tower Hamlets Clinical Commissioning Group (CCG), the Clinical Academic Group (CEG- Centre of Primary Care and Public Health, Barts and The London School of Medicine and Dentistry) and the GPs in Tower Hamlets for their support and participation in the study.

Funding

This work was supported by Arthritis Research UK (grant number 18678).

Ethics Committee Approval

The National Research Ethics Service (NRES) was contacted and confirmed in writing that ethical approval for the project was not required from the NHS research ethics committee (REC). Additionally, the Joint Research & Development office at Barts and The London was contacted and confirmed the same. Ethical approval was therefore granted by QMUL and permission to use the relevant data was also granted by the Governance Team of NHS North East London and The City.

For peer review only - http://bmjopen.bmj.com/site/about/guidelines.xhtml

Data sharing

The investigators will share data used in developing the results presented in this manuscript on request to the corresponding author.

For peer review only

For peer review only - http://bmjopen.bmj.com/site/about/guidelines.xhtml

References

1 Starfield B, Shi L. Policy relevant determinants of health: an international perspective. *Health Policy* 2002;60:201-218. doi:10.1016/s0168-8510(01)00208-1

2 Coulter A. Managing demand: Managing demand at the interface between primary and secondary care. *BMJ* 1998;316:1974-1976. doi:10.1136/bmj.316.7149.1974

3 Starfield B. Is primary care essential?. *The Lancet* 1994;344:1129-1133. doi:10.1016/s0140-6736(94)90634-3

4 Franks P, Clancy C, Nutting P. Gatekeeping Revisited — Protecting Patients from Overtreatment. *New England Journal of Medicine* 1992;327:424-429. doi:10.1056/nejm199208063270613

5 Parsons S, Ingram M, Clarke-Cornwell A et al. A Heavy Burden: the occurrence and impact of musculoskeletal conditions in the United Kingdom today. (Manchester eScholar - The University of Manchester). Escholar.manchester.ac.uk. 2011.https://www.escholar.manchester.ac.uk/uk-ac-man-scw:123774 (accessed 10 Nov 2018).

6 Statistics » Quarterly Hospital Activity Data. England.nhs.uk. 2018.https://www.england.nhs.uk/statistics/statistical-work-areas/hospital-activity/quarterly-hospital-activity/qar-data/ (accessed 10 Nov 2018).

7 Speed C, Crisp A. Referrals to hospital-based rheumatology and orthopaedic services: seeking direction. *Rheumatology* 2005;44:469-471. doi:10.1093/rheumatology/keh504

8 Elwyn G, Stott N. Avoidable referrals? Analysis of 170 consecutive referrals to secondary care. *BMJ* 1994;309:576-578. doi:10.1136/bmj.309.6954.576

9 Roland M, Porter R, Matthews J et al. Improving care: a study of orthopaedic outpatient referrals. *BMJ* 1991;302:1124-1128. doi:10.1136/bmj.302.6785.1124

10 The 2022 GP: Compendium of Evidence. Rcgp.org.uk. 2018.http://www.rcgp.org.uk/~/media/Files/Policy/A-Z-policy/The-2022-GP-Compendium-of-Evidence.ashx (accessed 10 Nov 2018).

11 Summerton N. Positive and negative factors in defensive medicine: a questionnaire study of general practitioners. *BMJ* 1995;310:27-29. doi:10.1136/bmj.310.6971.27

12 Franks P, Williams G, Zwanziger J et al. Why do physicians vary so widely in their referral rates?. *Journal of General Internal Medicine* 2000;15:163-168. doi:10.1046/j.1525-1497.2000.04079.x

13 Sullivan C, Omar R, Ambler G et al. Case-mix and variation in specialist referrals in general practice. *British Journal of General Practice* 2005;55:529-533.

14 Kassam H, Tzortziou Brown V, O'Halloran P et al. General practitioners' attitude to sport and exercise medicine services: a questionnaire-based survey. *Postgraduate Medical Journal* 2014;90:680-684. doi:10.1136/postgradmedj-2013-132245

15 Blank L, Baxter S, Woods H et al. What is the evidence on interventions to manage referral from primary to specialist non-emergency care? A systematic review and logic model synthesis. *Health Services and Delivery Research* 2015;3:1-430. doi:10.3310/hsdr03240

For peer review only - http://bmjopen.bmj.com/site/about/guidelines.xhtml

16 Wilkin D, Smith A. Variation in general practitioners' referral rates to consultants. *J R Coll Gen Pract* 1987;37:350-353.

17 O'Donnell C. Variation in GP referral rates: what can we learn from the literature?. *Family Practice* 2000;17:462-471. doi:10.1093/fampra/17.6.462

18 Foot C, Naylor C, Imison C. The quality of GP diagnosis and referral. Kingsfund.org.uk. 2010.https://www.kingsfund.org.uk/sites/default/files/field/field_document/quality-gp-diagnosis-referral-gq-inquiry-research-paper-mar11.pdf (accessed 10 Nov 2018).

19 Pawson R, Greenhalgh J, Brennan C. Demand management for planned care: a realist synthesis. *Health Services and Delivery Research* 2016;4:1-222. doi:10.3310/hsdr04020

20 Craig P, Dieppe P, Macintyre S et al. Developing and evaluating complex interventions. Mrc.ukri.org. 2008.https://mrc.ukri.org/documents/pdf/complex-interventions-guidance/ (accessed 10 Nov 2018).

21 The Magenta Book. GOV.UK. 2011.https://www.gov.uk/government/publications/the-magenta-book (accessed 10 Nov 2018).

22 Tower Hamlets JSNA Reference Group. Joint Strategic Needs Assessment Summary Document. Towerhamlets.gov.uk. 2016.https://www.towerhamlets.gov.uk/Documents/Public-Health/JSNA/JSNA_Summary.pdf (accessed 10 Nov 2018).

23 Michie S, Johnston M, Abraham C et al. Making psychological theory useful for implementing evidence based practice: a consensus approach. *Quality and Safety in Health Care* 2005;14:26-33. doi:10.1136/qshc.2004.011155

24 French S, Green S, O'Connor D et al. Developing theory-informed behaviour change interventions to implement evidence into practice: a systematic approach using the Theoretical Domains Framework. *Implementation Science* 2012;7. doi:10.1186/1748-5908-7-38

25 Porcheret M, Main C, Croft P et al. Development of a behaviour change intervention: a case study on the practical application of theory. *Implementation Science* 2014;9. doi:10.1186/1748-5908-9-42

26 Thomas S, Mackintosh S. Use of the Theoretical Domains Framework to Develop an Intervention to Improve Physical Therapist Management of the Risk of Falls After Discharge. *Physical Therapy* 2014;94:1660-1675. doi:10.2522/ptj.20130412

27 Sales A, Smith J, Curran G et al. Models, Strategies, and Tools. *Journal of General Internal Medicine* 2006;21:S43-S49. doi:10.1111/j.1525-1497.2006.00362.x

28 Roland M, McDonald R, Sibbald B. Can primary care reform reduce demand on hospital outpatient departments?. Netscc.ac.uk. 2007.http://www.netscc.ac.uk/hsdr/files/project/SDO_RS_08-1518-082_V01.pdf (accessed 10 Nov 2018).

29 Imison C, Naylor C. Referral management: Lessons for Success. Kingsfund.org.uk. 2010.https://www.kingsfund.org.uk/sites/files/kf/Referral-management-lessons-for-success-Candace-Imison-Chris-Naylor-Kings-Fund-August2010.pdf (accessed 10 Nov 2018).

30 Blundell N, Clarke A, Mays N. Interpretations of referral appropriateness by senior health managers in five PCT areas in England: a qualitative investigation. *Quality and Safety in Health Care* 2010;19:182-186. doi:10.1136/qshc.2007.025684

For peer review only - http://bmjopen.bmj.com/site/about/guidelines.xhtml

31 Stats Direct Statistical software. Stats Direct. https://www.statsdirect.co.uk/ (accessed 10 Nov 2018).

32 Open Exeter - NHS Digital. NHS Digital. https://digital.nhs.uk/NHAIS/open-exeter (accessed 10 Nov 2018).

33 Taljaard M, McKenzie J, Ramsay C et al. The use of segmented regression in analysing interrupted time series studies: an example in pre-hospital ambulance care. *Implementation Science* 2014;9. doi:10.1186/1748-5908-9-77

34 Ramsay C, Matowe L, Grilli R et al. INTERRUPTED TIME SERIES DESIGNS IN HEALTH TECHNOLOGY ASSESSMENT: LESSONS FROM TWO SYSTEMATIC REVIEWS OF BEHAVIOR CHANGE STRATEGIES. *International Journal of Technology Assessment in Health Care* 2003;19:613-623. doi:10.1017/s0266462303000576

35 Cochrane Effective Practice and Organisation of Care Review Group: Data Collection Checklist. Epoc.cochrane.org. http://epoc.cochrane.org/sites/epoc.cochrane.org/files/uploads/datacollectionchecklist.pdf (accessed 10 Nov 2018).

36 EPOC resources for review authors. Epoc.cochrane.org. http://epoc.cochrane.org/epoc-specific-resources-review-authors (accessed 10 Nov 2018).

37 Serumaga B, Ross-Degnan D, Avery A et al. Effect of pay for performance on the management and outcomes of hypertension in the United Kingdom: interrupted time series study. *BMJ* 2011;342:d108-d108. doi:10.1136/bmj.d108

38 Wagner A, Soumerai S, Zhang F et al. Segmented regression analysis of interrupted time series studies in medication use research. *Journal of Clinical Pharmacy and Therapeutics* 2002;27:299-309. doi:10.1046/j.1365-2710.2002.00430.x

39 Ajzen I. The theory of planned behavior. *Organizational Behavior and Human Decision Processes* 1991;50:179-211. doi:10.1016/0749-5978(91)90020-t

40 Rogers E. *Diffusion of Innovations*. 5th ed. Free Press 2003.

41 Mittman B, Tonesk X, Jacobson P. Implementing Clinical Practice Guidelines: Social Influence Strategies and Practitioner Behavior Change. *QRB - Quality Review Bulletin* 1992;18:413-422. doi:10.1016/s0097-5990(16)30567-x

42 Plsek P, Greenhalgh T. Complexity science: The challenge of complexity in health care. *BMJ* 2001;323:625-628. doi:10.1136/bmj.323.7313.625

43 Dawda P, Jenkins R, Varnam R. Quality improvement in general practice: An Inquiry into the Quality of General Practice in England. Kingsfund.org.uk. 2010.https://www.kingsfund.org.uk/sites/default/files/field/field_document/quality-improvement-gp-inquiry-discussion-paper-mar11.pdf (accessed 10 Nov 2018).

44 Evans E. The Torfaen referral evaluation project. *Quality in Primary Care* 2009;17:423-429.

45 Evans E, Aiking H, Edwards A. Reducing variation in general practitioner referral rates through clinical engagement and peer review of referrals: a service improvement project. *Qual Primary Care* 2018;19:263-272.

For peer review only - http://bmjopen.bmj.com/site/about/guidelines.xhtml

46 Briggs T. A national review of adult elective orthopaedic services in England: Getting right first time. Boa.ac.uk. 2015.https://www.boa.ac.uk/wp-content/uploads/2015/03/GIRFT-National-Report-MarN.pdf (accessed 10 Nov 2018).

47 Marjoribanks T, Lewis J. Reform and autonomy: perceptions of the Australian general practice community. *Social Science & Medicine* 2003;56:2229-2239. doi:10.1016/s0277-9536(02)00239-3

48 Spooner A, Chapple A, Roland M. What makes British general practitioners take part in a quality improvement scheme?. *Journal of Health Services Research & Policy* 2001;6:145-150. doi:10.1258/1355819011927396

49 Mead N, Roland M. Understanding why some ethnic minority patients evaluate medical care more negatively than white patients: a cross sectional analysis of a routine patient survey in English general practices. *BMJ* 2009;339:b3450-b3450. doi:10.1136/bmj.b3450

50 Campbell J, Ramsay J, Green J. Age, gender, socioeconomic, and ethnic differences in patients' assessments of primary health care. *Quality in Health Care* 2001;10:90-95. doi:10.1136/qhc.10.2.90

51 Choudhury Y, Bremner S, Ali A et al. Prevalence and impact of chronic widespread pain in the Bangladeshi and White populations of Tower Hamlets, East London. *Clinical Rheumatology* 2013;32:1375-1382. doi:10.1007/s10067-013-2286-3

52 Jamtvedt G, Young J, Kristoffersen D et al. Audit and feedback: Effects on professional practice and healthcare outcomes. *Physiotherapy* 2003;89:517. doi:10.1016/s0031-9406(05)60176-9

53 Bandura A. Self-efficacy: Toward a unifying theory of behavioral change. *Psychological Review* 1977;84:191-215. doi:10.1037//0033-295x.84.2.191

54 Watkins C, Timm A, Gooberman-Hill R et al. Factors affecting feasibility and acceptability of a practice-based educational intervention to support evidence-based prescribing: a qualitative study. *Family Practice* 2004;21:661-669. doi:10.1093/fampra/cmh614

55 Wright J, Harrison S, McGeorge M et al. Improving the management and referral of patients with transient ischaemic attacks: a change strategy for a health community. *Quality and Safety in Health Care* 2006;15:9-12. doi:10.1136/qshc.2005.014704

56 Tzortziou Brown V, Patel I, Thomas N et al. New ways of working; delivering better care for people with long-term conditions. *London Journal of Primary Care* 2017;9:60-68. doi:10.1080/17571472.2017.1361619

57 Gilburt H. Supporting integration through new roles and working across boundaries. Kingsfund.org.uk. 2016.https://www.kingsfund.org.uk/sites/files/kf/field/field_publication_file/Supporting_integration_web.pdf (accessed 10 Nov 2018).

58 Martin G, Kocman D, Stephens T et al. Pathways to professionalism? Quality improvement, care pathways, and the interplay of standardisation and clinical autonomy. *Sociology of Health & Illness* 2017;39:1314-1329. doi:10.1111/1467-9566.12585

59 Grol R, Grimshaw J. From best evidence to best practice: effective implementation of change in patients' care. *The Lancet* 2003;362:1225-1230. doi:10.1016/s0140-6736(03)14546-1

60 Sedgwick P. The Hawthorne effect. *BMJ* 2012;344:d8262-d8262. doi:10.1136/bmj.d8262

For peer review only - http://bmjopen.bmj.com/site/about/guidelines.xhtml

61 Squires J, Sullivan K, Eccles M et al. Are multifaceted interventions more effective than single-component interventions in changing health-care professionals' behaviours? An overview of systematic reviews. *Implementation Science* 2014;9. doi:10.1186/s13012-014-0152-6

62 Ivers N, Jamtvedt G, Flottorp S et al. Audit and feedback: effects on professional practice and healthcare outcomes. *Cochrane Database of Systematic Reviews* Published Online First: 2012. doi:10.1002/14651858.cd000259.pub3

63 Johnson M, May C. Promoting professional behaviour change in healthcare: what interventions work, and why? A theory-led overview of systematic reviews. *BMJ Open* 2015;5:e008592. doi:10.1136/bmjopen-2015-008592

64 Hall D, James D, Marsden N. Olympic lessons in high performance for organisations. *HR Bulletin: Research and Practice* 2012;7:9-13.

For peer review only

For peer review only - http://bmjopen.bmj.com/site/about/guidelines.xhtml

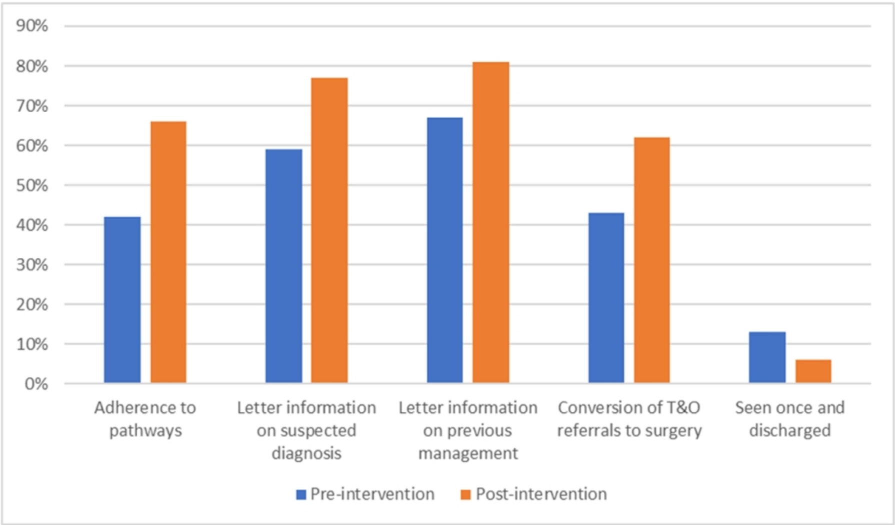

Summary of audit results on the quality of GP referrals

127x71mm (300 x 300 DPI)

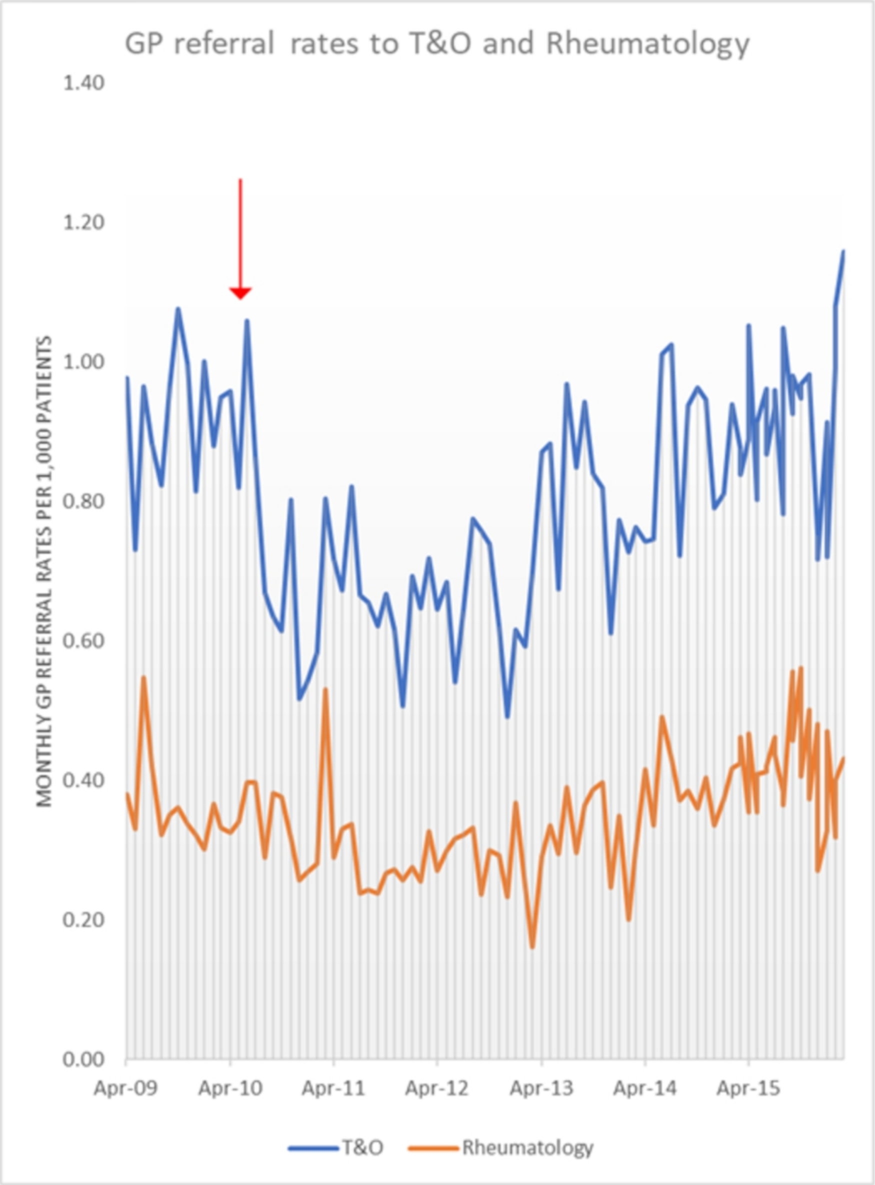

Number of GP referrals to T&O and Rheumatology per 1,000 patients between April 2009 and April 2016 (the red arrow represents the time point of the intervention, where T&O=Trauma and Orthopaedics)

139x190mm (300 x 300 DPI)

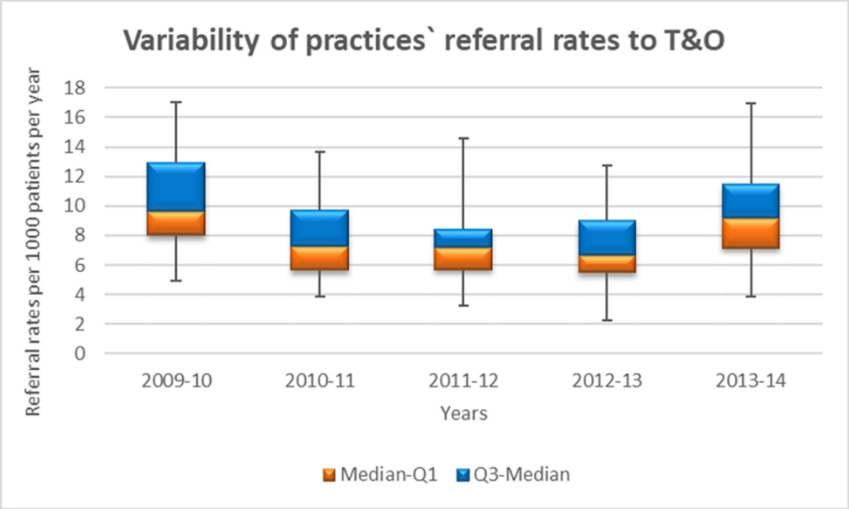

Multiple box and whisker plot showing the variability of practices` referral rates to Trauma and Orthopaedics (T&O)

127x71mm (300 x 300 DPI)

Revised Standards for Quality Improvement Reporting Excellence (SQUIRE 2.0) September 15, 2015
Text Section and Item Name Section or Item Description

	Check	Page
1. Title<br>Indicate that the manuscript concerns an initiative to improve healthcare (broadly defined to include the quality, safety, effectiveness, patient centeredness, timeliness, cost, efficiency, and equity of healthcare)	☑	1
The title of the manuscript is: “Improving the management of musculoskeletal conditions: can an alternative approach to referral management underpinned by quality improvement and behavioural change theories offer a solution and a better patient experience? A mixed methods study”		
2. Abstract<br>a. Provide adequate information to aid in searching and indexing b. Summarize all key information from various sections of the text using the abstract format of the intended publication or a structured summary such as: background, local problem, methods, interventions, results, conclusions.	☑	2
We have attempted to summarise the work in a succinct way in the abstract to assist with searching and indexing. We used the structure proposed in BMJ Open and included the objectives, design, setting and participants, intervention, main outcome measures, results and conclusion.		
3. Introduction<br>Why did you start? Problem Description Nature and significance of the local problem	☑	3
The significance of the topic has been justified in the introduction highlighting both the burden of MSK conditions and the challenges from increasing demand and number of outpatient referrals.		
4. Available knowledge Summary of what is currently known about the problem, including relevant previous studies	☑	3
The existing evidence has been summarised in the introduction and the gaps in evidence have been highlighted (e.g. limited theoretical underpinning of published interventions and insufficient evidence on effect on intermediate outcomes).		
5. Rationale<br>Informal or formal frameworks, models, concepts, and/or theories used to explain the problem, any reasons or assumptions that were used to develop the intervention(s), and reasons why the intervention(s) was expected to work	☑	4
The intervention had components targeting the main factors which influence clinical behaviour and behaviour change across the twelve domains described in the Theoretical Domains Framework (TDF). A detailed account of all the components of the intervention, how they link to the above theoretical domain framework and the supporting theories which underpin them has been presented in Table 1.		

For peer review only

6. Specific aims Purpose of the project and of this report<br>Methods What did you do?	☑	3-6
The aims and methodology of the project have been detailed in the manuscript (mixed methods including interrupted time series). We evaluated the implementation of a general practice focused intervention grounded in evidence synthesis underpinned by behavioural change theory, based on quality improvement (QI) principles and using mixed methodology. We used the MRC framework for complex interventions in the design, implementation, evaluation, and piloting phases of the multi-faceted intervention. The evaluation incorporated a process evaluation guided by HM Treasury`s guidance for evaluation 'The Magenta Book', and an outcomes evaluation which assessed the impact of the intervention on referral quality, referral rates, rate variability and patient experience.		
7. Context Contextual elements considered important at the outset of introducing the intervention(s)	☑	3-4
The context of the intervention has been described. Information has been provided on the setting of the intervention and the local availability of musculoskeletal service provision. The intervention was commissioned by the local Clinical Commissioning Group.		
8. Intervention(s)<br>a. Description of the intervention(s) in sufficient detail that others could reproduce it<br>b. Specifics of the team involved in the work	☑	4
The different elements of the intervention and the behavioural theories underpinning these have been described.<br>The intervention was co-ordinated by the local Clinical Commissioning Group. The principal investigator worked with clinical commissioners to collate and communicate all relevant referral data to practices at monthly intervals.		
9. Study of the Intervention(s)<br>a. Approach chosen for assessing the impact of the intervention(s)<br>b. Approach used to establish whether the observed outcomes were due to the intervention(s)	☑	5
A range of outcomes have been assessed and this is a strength of this study compared to other published reports which only focused on one or two relevant outcomes. The process evaluation included an assessment of how well each of the components of the intervention were implemented, including the level of GP practice participation in the clinical audit, educational meetings and outreach visits. The assessment was based on data from the clinical commissioning group and provider progress reports. The degree of GP participation, and the feedback and reflections of GP practices and networks in their reports were analysed and grouped thematically. The results provided a measure of participants` interaction with the intervention.		
10. Measures<br>a. Measures chosen for studying processes and outcomes of the intervention(s), including rationale for choosing them, their operational definitions, and their validity	☑	5-6

For peer review only

and reliability<br>b. Description of the approach to the ongoing assessment of contextual elements that contributed to the success, failure, efficiency, and cost<br>c. Methods employed for assessing completeness and accuracy of data		
The choice of measures, data collection and analysis as well as potential bias sources and limitations have been described. These are detailed in pages 5 and 6. For example with regards to the choice of measures on the quality of referrals we wrote: "To assess the impact on referral quality, we assessed three attributes as described by Blundell et al (31): necessity, quality of process, and destination. To determine necessity, we measured the adherence of referrals to the agreed local clinical pathways. We used the information contained in the referral letters as an indicator of quality of process. Finally, we calculated surgical conversion rates and the percentage of referrals that were seen once and discharged from the hospital without intervention and used these as indicators of the appropriateness of referral destination."<br>The process evaluation assisted towards an ongoing assessment of contextual elements.<br>The audit and patient questionnaires were piloted prior to implementation. All audit data from participating practices was entered in the meta-analysis and the assessment on quality was blinded against a pre-specified set of objective criteria. All outcomes on referral quality were reported.<br>The data on referral rates was collected by an independent source (National Hospital Episode Statistics extract data) which was blind to the intervention. The seasonality of the data was tested using two-way ANOVA. If the results showed significant monthly variation, seasonal decomposition was used (IBM SPSS Statistics Version 22 software). The seasonally adjusted series (SAS) was entered in the ARIMA analysis.		
11. Analysis<br>a. Qualitative and quantitative methods used to draw inferences from the data<br>b. Methods for understanding variation within the data, including the effects of time as a variable	☑	5-6
These have been described in the methodology section. For example, with regards to the analysis of referral rates we wrote:<br>"We used an interrupted Time Series (ITS) analysis to evaluate the impact of the intervention on referral rates. ITS is a powerful quasi-experimental design for evaluating effects of interventions when random assignment is not feasible and is well suited to initial evaluations of community interventions with greater use of this method advocated for community intervention research. This design was particularly useful for capitalising on existing data on GP referrals collected at CCG level. In order to limit the risk of bias, the quality criteria for ITS design as published by Effective Practice and Organisation of Care (EPOC) Cochrane group were followed. Analysis using segmented time series regression technique (ARIMA- autoregressive integrated moving average) was used to control for baseline level and trend, a common approach for evaluating policy and educational interventions.<br><br>The analysis was undertaken using SPSS (Version 22, IBM). Relative effects were calculated using the predicted values from the ARIMA analysis. The seasonality of the data was tested using two-way ANOVA. If the results showed significant monthly variation, seasonal decomposition was used (IBM SPSS Statistics software, Version		

For peer review only

22) and the seasonally adjusted series (SAS) was entered in the ARIMA analysis."		
12. Ethical Considerations<br>Ethical aspects of implementing and studying the intervention(s) and how they were addressed, including, but not limited to, formal ethics review and potential conflict(s) of interest	☑	11
The study was considered by the relevant ethic committees and we have submitted information on the relevant approvals obtained.<br>"The National Research Ethics Service (NRES) was contacted and confirmed in writing that ethical approval for the project was not required from the NHS research ethics committee (REC). Additionally, the Joint Research & Development office at Barts and The London was contacted and confirmed the same. Ethical approval was therefore granted by QMUL and permission to use the relevant data was also granted by the Governance Team of NHS North East London and The City."		
13. Results<br>a. Initial steps of the intervention(s) and their evolution over time (e.g., time-line diagram, flow chart, or table), including modifications made to the intervention during the project b. Details of the process measures and outcome c. Contextual elements that interacted with the intervention(s) d. Observed associations between outcomes, interventions, and relevant contextual elements e. Unintended consequences such as unexpected benefits, problems, failures, or costs associated with the intervention(s). f. Details about missing data	☑	6-9
The results have been summarised across all outcomes. No modifications were made to the intervention over time. A process evaluation took place and was reported. The contextual elements were reported as part of the process evaluation. Missing data was reported.		
14. Summary a. Key findings, including relevance to the rationale and specific aims b. Particular strengths of the project	☑	9-10
The key findings and strengths of the study have been presented under Discussion and Strengths and Limitations of the study. The main strengths and limitations have also been summarised in page 2.		
15. Interpretation<br>a. Nature of the association between the intervention(s) and the outcomes<br>b. Comparison of results with findings from other publications<br>c. Impact of the project on people and systems<br>d. Reasons for any differences between observed and anticipated outcomes, including the influence of context<br>e. Costs and strategic trade-offs, including opportunity costs	☑	9-10
The association between the intervention and the outcomes was explored using the relevant behavioural theories underpinning the intervention. Comparisons of the findings of this study with existing literature have been made in the discussion section. The impact of the project on participants was reported as part of the process		

For peer review only

evaluation. The lack of sustainability of the effect on referral rates could be explained by Bandura`s Social Cognitive Theory which highlights the importance of stimuli and reinforcement for learning and behavioural change. A cost-effectiveness or cost analysis was not undertaken.		
16. Limitations<br>a. Limits to the generalizability of the work<br>b. Factors that might have limited internal validity such as confounding, bias, or imprecision in the design, methods, measurement, or analysis<br>c. Efforts made to minimize and adjust for limitations	☑	3,10
The limitations of the study have been articulated and reflected upon.<br>For example, the last section mentions: "One of the study`s limitations was the fact that the design was by necessity non-randomised. The effect of the intervention may be partly attributable to increased attention, focus and priority on referral behaviour." The methodology section expanded on the choice of the design and the reasons for this choice as well as the attempts made to address confounding and bias.		
17. Conclusions<br>a. Usefulness of the work<br>b. Sustainability<br>c. Potential for spread to other contexts<br>d. Implications for practice and for further study in the field<br>e. Suggested next steps<br>Other information	☑	10
The usefulness of the work, generalisability and implications of the study have been summarised in the conclusions. The sustainability of the intervention was explored as the study had a long follow up rate. The potential next steps are summarised in the last paragraph. The developing more integrated systems of care and the technological advances offer opportunities for building on professionalism and further improving referral processes through inter-professional co-operation.		
18. Funding<br>Sources of funding that supported this work. Role, if any, of the funding organization in the design, implementation, interpretation, and reporting	☑	11
The funder (Arthritis Research UK) has been mentioned.		
